# Skull Development, Ossification Pattern, and Adult Shape in the Emerging Lizard Model Organism *Pogona vitticeps*: A Comparative Analysis With Other Squamates

**DOI:** 10.3389/fphys.2018.00278

**Published:** 2018-03-28

**Authors:** Joni Ollonen, Filipe O. Da Silva, Kristin Mahlow, Nicolas Di-Poï

**Affiliations:** ^1^Program in Developmental Biology, Institute of Biotechnology, University of Helsinki, Helsinki, Finland; ^2^Museum für Naturkunde, Leibniz Institute for Evolution and Biodiversity Science, Berlin, Germany

**Keywords:** *Pogona vitticeps*, lizard, squamate, embryogenesis, skull, model organism

## Abstract

The rise of the Evo-Devo field and the development of multidisciplinary research tools at various levels of biological organization have led to a growing interest in researching for new non-model organisms. Squamates (lizards and snakes) are particularly important for understanding fundamental questions about the evolution of vertebrates because of their high diversity and evolutionary innovations and adaptations that portrait a striking body plan change that reached its extreme in snakes. Yet, little is known about the intricate connection between phenotype and genotype in squamates, partly due to limited developmental knowledge and incomplete characterization of embryonic development. Surprisingly, squamate models have received limited attention in comparative developmental studies, and only a few species examined so far can be considered as representative and appropriate model organism for mechanistic Evo-Devo studies. Fortunately, the agamid lizard *Pogona vitticeps* (central bearded dragon) is one of the most popular, domesticated reptile species with both a well-established history in captivity and key advantages for research, thus forming an ideal laboratory model system and justifying his recent use in reptile biology research. We first report here the complete post-oviposition embryonic development for *P. vitticeps* based on standardized staging systems and external morphological characters previously defined for squamates. Whereas the overall morphological development follows the general trends observed in other squamates, our comparisons indicate major differences in the developmental sequence of several tissues, including early craniofacial characters. Detailed analysis of both embryonic skull development and adult skull shape, using a comparative approach integrating CT-scans and gene expression studies in *P. vitticeps* as well as comparative embryology and 3D geometric morphometrics in a large dataset of lizards and snakes, highlights the extreme adult skull shape of *P. vitticeps* and further indicates that heterochrony has played a key role in the early development and ossification of squamate skull bones. Such detailed studies of embryonic character development, craniofacial patterning, and bone formation are essential for the establishment of well-selected squamate species as Evo-Devo model organisms. We expect that *P. vitticeps* will continue to emerge as a new attractive model organism for understanding developmental and molecular processes underlying tissue formation, morphology, and evolution.

## Background

The wide recognition of the importance to connect the field of evolution with developmental biology (Evo-Devo) in a mechanistic perspective, alongside with both the development of new multidisciplinary research tools and the employment of new types of molecular data in modern comparative developmental approaches have led to an explosion of interest for new model organisms. In particular, squamate reptiles (lizard and snakes) are becoming popular animal models among evolutionary developmental biologists because of their high morphological and genetic diversity as well as exceptional anatomical and physiological innovations. Squamate models are particularly useful for shedding light onto the multiple genotype-phenotype paths taken in the evolution of amniotes and for understanding fundamental questions about the evolution of morphological diversity, including extreme body form such as limb loss and axial skeleton elongation (Gomez et al., 2008; Di-Poï et al., 2010; Head and Polly, 2015; Kvon et al., 2016; Leal and Cohn, 2016). For these purposes, developmental staging tables based on external embryonic features have been reported for multiple lizard and snake species from different families (Tables 1, 2). These staging tables are indispensable tools to describe the timing of developmental events in a specific taxon, and can be directly used for the comparison of developmental dynamics across species. Such comparative studies in squamates have expanded the comparative bases of tooth, axial skeleton, skull, limb, and genital development (Tables 1, 2), and have played an important role for establishing evolutionary patterns among vertebrates. In many cases, however, the staging tables are fragmented and do not cover all external features and/or the whole developmental period of particular squamate species; as a consequence, complete tables exist for only a small sample of lizard and snake species (Tables 1, 2). In addition, whereas the complete intra-uterine development of *Zootoca vivipara* (Dufaure and Hubert, 1961) and the more recent Standard Event System (SES) developed by Werneburg (2009) have been adopted as the preferred references for comparisons of embryonic characters (Wise et al., 2009; Roscito and Rodrigues, 2012a,b; Polachowski and Werneburg, 2013; Werneburg et al., 2015; Melville et al., 2016; Rapp Py-Daniel et al., 2017), many studies have used other staging tables or even independent staging criteria. Importantly, the latter studies rarely consider the presence of heterochronic shifts at different levels of organogenesis, in contrast to the standardized SES system, thus making embryonic comparisons between squamate species extremely challenging (Werneburg, 2009).

**Table 1:** Summary of complete and incomplete embryonic staging data available for lizards.

**Group**	**Family**	**Species**	**Embryonic stage**	**References**
Anguimorpha	Anguidae	*Anguis fragilis*	Incomplete (early stages)	Nicolas, 1904; Ballowitz, 1905; Meyer, 1910
Anguimorpha	Anguidae	*Barisia imbricata*	Incomplete (genital)	Martínez-Torres et al., 2015
Anguimorpha	Varanidae	*Varanus indicus*	Post-oviposition	Gregorovicova et al., 2012
Anguimorpha	Varanidae	*Varanus panoptes*	Post-oviposition	Werneburg et al., 2015
Gekkota	Eublepharidae	*Eublepharis macularius*	Post-oviposition	Wise et al., 2009
Gekkota	Gekkonidae	*Cyrtodactylus pubisulcus*	Incomplete (whole skeleton)	Rieppel, 1992a
Gekkota	Gekkonidae	*Gehyra oceanica*	Incomplete (limb skeleton)	Rieppel, 1994a
Gekkota	Gekkonidae	*Hemidactylus turcicus*	Incomplete (vertebrae)	Werner, 1971
Gekkota	Gekkonidae	*Lepidodactylus lugubris*	Incomplete (limb skeleton)	Rieppel, 1994a
Gekkota	Gekkonidae	*Paroedura pictus*	Post-oviposition	Noro et al., 2009
Gekkota	Gekkonidae	*Ptyodactylus hasselquistii*	Incomplete (vertebrae)	Werner, 1971
Gekkota	Phyllodactylidae	*Tarentola annularis*	Post-oviposition	Khannoon, 2015
Gekkota	Sphaerodactylidae	*Sphaerodactylus argus*	Incomplete (vertebrae)	Werner, 1971
Gekkota	Sphaerodactylidae	Sphaerodactyls	Post-oviposition (incomplete)	Guerra-Fuentes et al., 2014
Iguania	Agamidae	*Agama impalearis*	Pre- and post-oviposition	El Mouden et al., 2000
Iguania	Agamidae	*Calotes versicolor*	Post-oviposition	Muthukkarruppan et al., 1970
Iguania	Agamidae	*Calotes versicolor*	Pre-oviposition	Thapliyal et al., 1973
Iguania	Agamidae	*Pogona vitticeps*	Incomplete (limb)	Melville et al., 2016
Iguania	Agamidae	*Pogona vitticeps*	Incomplete (limb, genital)	Whiteley et al., 2017
Iguania	Chamaeleonidae	*Chamaeleo bitaeniatus*	Post-oviposition	Pasteels, 1956
Iguania	Chamaeleonidae	*Chamaeleo calyptratus*	Post-oviposition (incomplete)	Andrews, 2007
Iguania	Chamaeleonidae	*Chamaeleo hoehnelii*	Incomplete (whole skeleton)	Rieppel, 1993
Iguania	Chamaeleonidae	*Chamaeleo lateralis*	Pre- and post-oviposition	Blanc, 1974
Iguania	Dactyloidae	*Anolis sagrei*	Pre- and post-oviposition	Sanger et al., 2008
Iguania	Liolaemidae	*Liolaemus tenuis*	Pre- and Post-oviposition	Lemus and Duvauchelle, 1966; Lemus et al., 1981
Iguania	Liolaemidae	*Liolaemus gravenhorstii*	Pre- and post-oviposition	Lemus, 1967
Iguania	Liolaemidae	*Liolaemus quilmes*	Incomplete (whole skeleton)	Abdala et al., 1997
Iguania	Liolaemidae	*Liolaemus scapularis*	Incomplete (whole skeleton)	Lobo et al., 1995
Iguania	Phrynosomatidae	*Uta stansburiana*	Post-oviposition (incomplete)	Andrews and Greene, 2011
Iguania	Polychrotidae	*Polychrus acutirostris*	Incomplete (whole skeleton)	Álvarez et al., 2005
Iguania	Tropiduridae	*Tropidurus etheridgei*	Incomplete (whole skeleton)	Lions and Alvarez, 1998
Iguania	Tropiduridae	*Tropidurus torquatus*	Post-oviposition	Rapp Py-Daniel et al., 2017
Lacertoidea	Amphisbaenidae	*Amphisbaena darwini*	Incomplete (skull)	Montero et al., 1999
Lacertoidea	Gymnophthalmidae	*Calyptommatus nicterus*	Incomplete (skull)	Roscito and Rodrigues, 2010
Lacertoidea	Gymnophthalmidae	*Calyptommatus sinebrachiatus*	Post-oviposition	Roscito and Rodrigues, 2012a
Lacertoidea	Gymnophthalmidae	*Nothobachia ablephara*	Post-oviposition	Roscito and Rodrigues, 2012b
Lacertoidea	Gymnophthalmidae	*Nothobachia ablephara*	Incomplete (skull)	Roscito and Rodrigues, 2010
Lacertoidea	Gymnophthalmidae	*Ptychoglossus bicolor*	Incomplete (skull)	Hernandez-Jaimes et al., 2012
Lacertoidea	Gymnophthalmidae	*Scriptosaura catimbau*	Incomplete (skull)	Roscito and Rodrigues, 2010
Lacertoidea	Lacertidae	*Lacerta agilis*	Pre- and post-oviposition	Peter, 1904
Lacertoidea	Lacertidae	*Lacerta agilis*	Incomplete (skull)	Rieppel, 1994b
Lacertoidea	Lacertidae	*Lacerta viridis*	Post-oviposition	Dhouailly and Saxod, 1974
Lacertoidea	Lacertidae	*Podarcis muralis*	Post-oviposition	Dhouailly and Saxod, 1974
Lacertoidea	Lacertidae	*Zootoca vivipara*	Intra-uterine	Dufaure and Hubert, 1961
Lacertoidea	Lacertidae	*Zootoca vivipara*	Incomplete (skull)	Rieppel, 1992b
Lacertoidea	Teiidae	*Tupinambis* sp.	Incomplete (whole skeleton)	Arias and Lobo, 2006
Scincoidea	Scincidae	*Hemiergis* sp.	Incomplete (limb)	Shapiro, 2002
Scincoidea	Scincidae	*Liopholis whitii*	Incomplete (whole skeleton)	Hugi et al., 2010
Scincoidea	Scincidae	*Mabuya capensis*	Incomplete (skull)	Skinner, 1973
Scincoidea	Scincidae	*Trachylepis megalura*	Intra-uterine	Pasteels, 1956

*Complete staging tables are highlighted with gray shading*.

**Table 2:** Summary of complete and incomplete embryonic staging data available for snakes.

**Group**	**Family**	**Species**	**Embryonic stage**	**References**
Serpentes	Achrochordidae	*Acrochordus granulatus*	Incomplete (skull)	Rieppel and Zaher, 2001
Serpentes	Colubridae	*Elaphe quadrivirgata*	Pre-oviposition	Matsubara et al., 2014
Serpentes	Colubridae	*Natrix natrix*	Post-oviposition	Krull, 1906; Vielhaus, 1907
Serpentes	Colubridae	*Natrix natrix*	Incomplete (skull)	Kovtun and Sheverdyukova, 2015
Serpentes	Colubridae	*Natrix tessellata*	Post-oviposition	Korneva, 1969
Serpentes	Colubridae	*Crotaphopeltis hotamboeia*	Incomplete (skull)	Brock, 1929
Serpentes	Colubridae	*Nerodia taxispilota*	Incomplete (skull)	Franklin, 1945
Serpentes	Colubridae	*Pantherophis obsoletus*	Incomplete (skull)	Haluska and Alberch, 1983
Serpentes	Colubridae	*Psammophis sibilans*	Post-oviposition	Khannoon and Zahradnicek, 2016
Serpentes	Colubridae	*Thamnophis sirtalis*	Intra-uterine	Zehr, 1962
Serpentes	Elapidae	*Naja haje*	Post-oviposition	Khannoon and Evans, 2013
Serpentes	Elapidae	*Naja haje*	Incomplete (skull)	Khannoon and Evans, 2015
Serpentes	Elapidae	*Naja kaouthia*	Post-oviposition	Jackson, 2002
Serpentes	Lamprophiidae	*Boaedon fuliginosus*	Post-oviposition	Boback et al., 2012
Serpentes	Pythonidae	*Python sebae*	Post-oviposition	Boughner et al., 2007
Serpentes	Pythonidae	*Python sebae*	Incomplete (histology)	Buchtova et al., 2007
Serpentes	Viperidae	*Agkistrodon piscivorus*	Incomplete (skull)	Savitzky, 1992
Serpentes	Viperidae	*Bothropoides jararaca*	ost-oviposition	Polachowski and Werneburg, 2013
Serpentes	Viperidae	*Vipera aspis*	Intra-uterine	Hubert and Dufaure, 1968

*Complete staging tables are highlighted with gray shading*.

While previous embryonic descriptions of squamates have largely focused on non-venomous and oviparous species, only a few species examined so far can be considered as representative and appropriate model organism for detailed Evo-Devo studies. In particular, a lack of developmental information for agamid lizards (Agamidae) is particularly surprising given the fact that they are a widespread, diverse iguanian group with a well-known ecology, life-history, and behavior (Witten, 1993; Zoffer and Mazorlig, 1997; Doneley, 2006; Cogger, 2014). At the developmental level, complete staging tables for both pre- and post-oviposition development have been reported for two agamid species, *Agama impalearis* (El Mouden et al., 2000) and *Calotes versicolor* (Muthukkarruppan et al., 1970). However, incomplete developmental information is available for some more popularly known, domesticated species such as the central bearded dragon *Pogona vitticeps*, with only a few recent reports focusing on limb and/or genital development using different non-standardized staging systems (Melville et al., 2016; Whiteley et al., 2017). *P. vitticeps* has been increasingly used in recent reptile biology research (see, e.g., Tzika et al., 2015; Di-Poï and Milinkovitch, 2016; Melville et al., 2016; Whiteley et al., 2017) because this species forms an ideal laboratory model system to examine developmental and molecular processes underlying tissue morphology and evolution. This model has several unique characteristics shared by the genus *Pogona* and relevant for scientific research, including e.g., relatively large size, broad triangular head, flattened bodies, heterogeneous body scales with elongated spiny scales, variable skin coloration, acrodont teeth, chromosomal- or temperature-dependent sex determination, and primitive venom glands (Witten, 1993; de Vosjoli and Maillou, 1996; Zoffer and Mazorlig, 1997; Ezaz et al., 2005; Fry et al., 2006; Cogger, 2014; Whiteley et al., 2017). Similarly to other oviparous lizard models proposed for Evo-Devo studies—the leopard gecko *Eublepharis macularius* (Vickaryous and McLean, 2011), the genus *Anolis* (Sanger et al., 2008), and the veiled chameleon *Chamaeleo calyptratus* (Diaz and Trainor, 2017)— *P. vitticeps* has a well-established history in captivity and hatchlings are easily raised to adulthood, enabling breeding lines to be established. For examples, a variety of color morphs and scaleless *Eda*-mutants already exists as popular pets (de Vosjoli and Maillou, 1996; Di-Poï and Milinkovitch, 2016). Importantly, *P. vitticeps* also combines several key advantages only present in some of the other proposed models, including easy animal availability, docile behavior (Zoffer and Mazorlig, 1997), available reference genome and transcriptome data (Georges et al., 2015; Tzika et al., 2015), large clutches of eggs (up to 35 eggs) several times per season (Köhler, 2005), and relatively short post-oviposition incubation period (about 60 days at “normal” temperature; Köhler, 2005; Whiteley et al., 2017). Finally, the laying of relatively large eggs in *P. vitticeps* is an attractive feature for performing *in ovo* microsurgical and/or genetic manipulations of developing embryos (Hull et al., 2014; Nomura et al., 2015).

Among squamates, agamids are a successful group of lizards, with a great diversity in habitat, diet, biome, and morphology. They comprise more than 450 species, widely distributed across hot deserts and tropical rainforests, with diverse dietary preferences such as insectivorous, myrmecophagous, omnivorous, and herbivorous. In addition, agamid lizards have experienced independent radiations with remarkable life-history strategy and phenotypic diversity in terms of body form and size as well as coloration, pholidosis, and dental patterns across different continents, including Australia, Africa, Asia, and Europe. Such extraordinary morphological and ecological diversity makes agamids an excellent group to elucidate the mechanisms that promote lineage diversification and phenotypic variation at different taxonomic levels both within and among squamate population and species. The key research advantages of the *P. vitticeps* could be further used for testing and confirming the role of developmental processes and pathways in shaping and constraining the phenotypic diversity and for revealing the processes by which phenotypic variation arise. Similarly, interesting patterns of convergent ecological and morphological evolution have already been identified in agamids (Melville et al., 2006), and comparative, mechanistic Evo-Devo studies in several agamid lizards including *P. vitticeps* could help understanding the evolution of morphological traits and processes such as convergence, allometry, and evolution of growth in related organism. Such understanding of the mechanisms underlying the development of morphological diversity in lizards will also have wider importance across vertebrates, in particular for studies of the complex association between ecology and evolution in mammals.

As a prerequisite for the establishment and use of new reptile species as Evo-Devo model organisms, we describe here the complete, detailed post-oviposition development of the agamid lizard *P. vitticeps* based on external morphological characters previously defined for squamates, using the standardized SES staging system (Werneburg, 2009; Polachowski and Werneburg, 2013; Werneburg et al., 2015) and other commonly used staging characters available for snake and lizard species (Dufaure and Hubert, 1961; Sanger et al., 2008). In addition to external morphological development, we describe in more detail the early patterning, cranial ossification pattern, and adult shape of skull bones, using a complementary approach integrating micro-computed tomography (CT) scans, 3D geometric morphometrics, comparative embryology, and gene expression studies. The cranial skeleton is one of the most diversified bony structures of squamate reptiles (Evans, 2008; Werneburg and Sánchez-Villagra, 2015; Da Silva et al., 2018), and investigation of craniofacial development and ossification events are important in understanding the ontogenetic processes behind morphological diversity and ecological adaptation, but also in elucidating phylogenetic relationships on different taxonomic levels. In addition, an easily available, complete morphological description of the embryonic skull development is still lacking for most lizard families, including agamids, and despite the recent growing interest for cranial evolution and development in different vertebrate species, there has been limited comparative work and discussion on skull development among lizards.

## Materials and methods

### Collection of eggs and embryos

All of the *P. vitticeps, Anolis carolinensis, Boaedon fuliginosus*, and *Pantherophis guttatus* eggs were obtained from our animal facility at the University of Helsinki. Eggs from *Lampropeltis getula, Python sebae*, and *Furcifer pardalis* were obtained from reptile breeders or Tropicario Helsinki. Fertilized eggs were incubated on a moistened vermiculite substrate at 29.5°C (or 26°C for *F. pardalis*) until opened. *P. vitticeps* embryos were collected from different females and over three breeding seasons to obtain consistent and accurate staging. Early and/or small embryos were imaged using a stereomicroscope (Zeiss SteREO Lumar V12, Objective: ApoLumar S 1.2 FWD 47 mm, Camera: Axiocam ICc 1) to create z-stacks, and stacks were combined and converted into TIFF files using Zeiss Zen 2.3 (Blue edition). Larger embryos were imaged using a Nikon D3200 digital camera and incident light. Photographs of other embryonic species were sampled from published literature.

### CT-scanning and 3D rendering

CT-scans of adult skulls (cranium and mandible) covering all major lineages of squamates (Additional Files 1, 2) were primarily obtained from the publicly available Digital Morphology Database (DigiMorph) or from our previous work (Da Silva et al., 2018). New high-resolution CT-scans of embryonic and adults skulls were produced at the University of Helsinki imaging facility using Skyscan 1272 microCT or Phoenix Nanotom 180, depending on the specimen size. To visualize skull bone development, fixed *P. vitticeps* embryos were CT-scanned at different days post-oviposition (15, 18, 24, 28, 32, 36, 40, 48, and 60 dpo) using the following parameters: filter: Al 0.25 mm; voltage: 60 kV; current: 166 μA; resolution: 6 μm. Scans were reconstructed using Bruker NRecon 1.7.0.4 software, and 3D isosurface rendering as well as segmentation of cranial bones were done using a variety of density thresholds with the software Amira 5.5.0 (Visualization Sciences Group). All 3D data were scaled by voxel size based on scan log file in Amira 5.5.0. For overall visualization of soft tissue and comparative morphology at early embryonic stages, embryos were first stained with 0.6% phosphotungstic acid (PTA) in ethanol for 14 days, as described before (Metscher, 2009), before scanning using the following parameters: filter: Al 1 mm; voltage: 80 kV; current: 125 μA; resolution: 6 μm.

### General measurements and staging criteria

More than 140 *P. vitticeps* embryos taken at different developmental stages were analyzed. Measurements were done on photographs using a ruler and CorelDRAW X8. Snout-vent length (SVL) and total length (TL) were measured from snout to cloaca and from snout to tip of the tail, respectively, using the lateral profile of embryos. The measurements had a variance of ±1 mm. Staging of *P. vitticeps* embryos was performed from oviposition to hatchling based on the Standard Event System (SES) using external morphological characters previously defined for squamates (Werneburg, 2009; Polachowski and Werneburg, 2013; Werneburg et al., 2015; see also Additional File 3), but also with the help of complete staging tables available for additional lizard species such as *Z. vivipara* and *Anolis sagrei* (Dufaure and Hubert, 1961; Sanger et al., 2008). Detailed external morphological characters in *P. vitticeps* at different days post-oviposition were predominantly mapped on intact, freshly dissected embryos using a Zeiss SteREO Lumar V12 stereomicroscope, but high-resolution CT-scans of embryos stained with the contrast agent PTA were also used to visualize soft tissue details in 3D (Metscher, 2009). The main external characters employed in our series include the number of somites, the number of pharyngeal arches and slits, as well as the developmental level of the neural tube, head, nose, ear, eye, and accessory visual structures, rib primordia, heart, limbs, scales, facial prominences, urogenital papillae, neck, and hemipenes (Additional File 3). Statements concerning carapace scutes and the caruncle of the eye were not possible because the structures were not present in lizards or could not be properly observed, respectively. Other traits not considered in the SES staging system but recently described (Whiteley et al., 2017), including the projections of the developing brain and the appearance of pigmentation, were also used as characteristics when staging with the developmental tables of *Z. vivipara* and *A. sagrei* (Table 1).

### 3D geometric morphometrics and multivariate statistics

The shape of the cranium and mandible was extracted separately in 112 adult squamate species (Additional File 2) by digitizing 65 and 18 landmarks, respectively, in the Stratovan package Checkpoint (Additional Files 4–7). The definition of our landmarks for the cranium followed the terminology described previously for squamates (Da Silva et al., 2018), and additional landmarks were positioned on the mandible, palate, vomer, and prootic bone (Additional Files 4–6). Bone descriptions follow the terminology and skull regions previously defined for squamates (Evans, 2008). Bones absent in different squamate lineages such as the temporal bar, coronoid, ectopterygoid, septomaxilla, and supratemporal bones were not included. Data were scaled, translated, and oriented via a generalized Procrustes analysis (GPA) superimposition method (Klingenberg, 2010). The evolutionary patterns of both cranium and mandible shape disparity were visualized using a principal component analysis (PCA) as implemented in the package MorphoJ v1.06 (Klingenberg, 2011). This method summarizes the multidimensional shape data through independent orthogonal axes of main shape variation. The influence of size or evolutionary allometry was tested using a multivariate regression analysis of independent-contrasts of shape (Procrustes coordinates) on size (centroid size) (Monteiro, 1999). For assessing shape evolution in a phylogenetic context, a phylomorphospace was generated by first plotting the main PC scores on the most-inclusive phylogenetic tree for squamates (Tonini et al., 2016), and then by reconstructing the ancestral shapes of the internal nodes using weighted squared-change parsimony algorithms (Maddison, 1991) in MorphoJ v1.06. Phylogenetic signal was calculated using a multivariate generalized *K*-statistic (Adams, 2014) in the R-package geomorph v3.0.5 (Adams and Otárola-Castillo, 2013). Non-parametric multivariate analysis of variance (*MANOVA*) with Bonferroni-corrected *post-hoc* pairwise comparisons were used to test for significant shape differences (based on 10,000 permutations) between major groups of squamates in the software PAST v.3.18 (Hammer et al., 2001). Dibamia could not be used because of the presence of only one species in our dataset (Additional File 2). Comparisons were made based on the first 10 PCs, accounting for >90% of total shape variation. Shape diagrams for the extreme values of the principal components (PCs) were depicted with the thin-plate spline (TPS) technique in MorphoJ v1.06 and color-coded according to major skull regions.

### Whole-mount *in situ* hybridization and immunofluorescence

*P. vitticeps* embryos at oviposition were fixed overnight in 4% paraformaldehyde (PFA) in PBS at 4°C, dehydrated though a series of methanol/PBS solutions (25, 50, 75, and 100% methanol), and stored at −20°C until hybridization or immunofluorescence. Whole mount *in situ* hybridization (WMISH) was performed according to our previously published protocol (Di-Poï et al., 2010) at a temperature of 68°C. New species-specific digoxigenin-labeled antisense riboprobes corresponding to *P. vitticeps Dlx2* (831 bp, 3′ UTR region) and *Sox10* (837 bp, 3′ UTR region) genes were designed based on publicly available *P. vitticeps* genome sequence (Georges et al., 2015). Corresponding sense riboprobes were used as negative controls. For immunofluorescence, embryos were embedded in paraffin following alcohol dehydration and then sectioned at 7 μm. Staining was performed as previously described (Di-Poï and Milinkovitch, 2013) using heat-induced epitope retrieval, primary antibodies known to recognize reptile and/or chicken epitopes (anti-β-tubulin: 1:400, Thermo Fischer Scientific; anti-ISLET-1: 1:700, Abcam), and Alexa Fluor-conjugated secondary antibodies (Alexa Fluor-488 or−568, Life Technologies). Samples were mounted with Fluoroshield mounting medium (Sigma) containing 4′,6′-diamidino-2-phenylindole (DAPI).

### Comparative analysis of early embryonic facial morphogenesis

A discrete scale approach was used to rank the degree of development of facial prominences in snake and lizard embryos at the oviposition stage (0–1 dpo). Six conspicuous early facial traits showing major developmental changes in morphology and/or growth were selected (see Additional File 9): shape of frontonasal prominence, length of maxillary prominence, shape of oral commissure (ventral bending in the proximal region of the first pharyngeal arch), presence/absence of mandible basal constriction, length of mandibular prominence, and level of fusion and differentiation of pharyngeal pouches between pharyngeal arches. States reflect a causal and temporal relationship between early and late events in the ontogeny, so that the appearance of a late trait is dependent of the appearance of an early trait during development (Albrech, 1985).

## Results

### External morphology and embryonic staging

**Figure 1:**
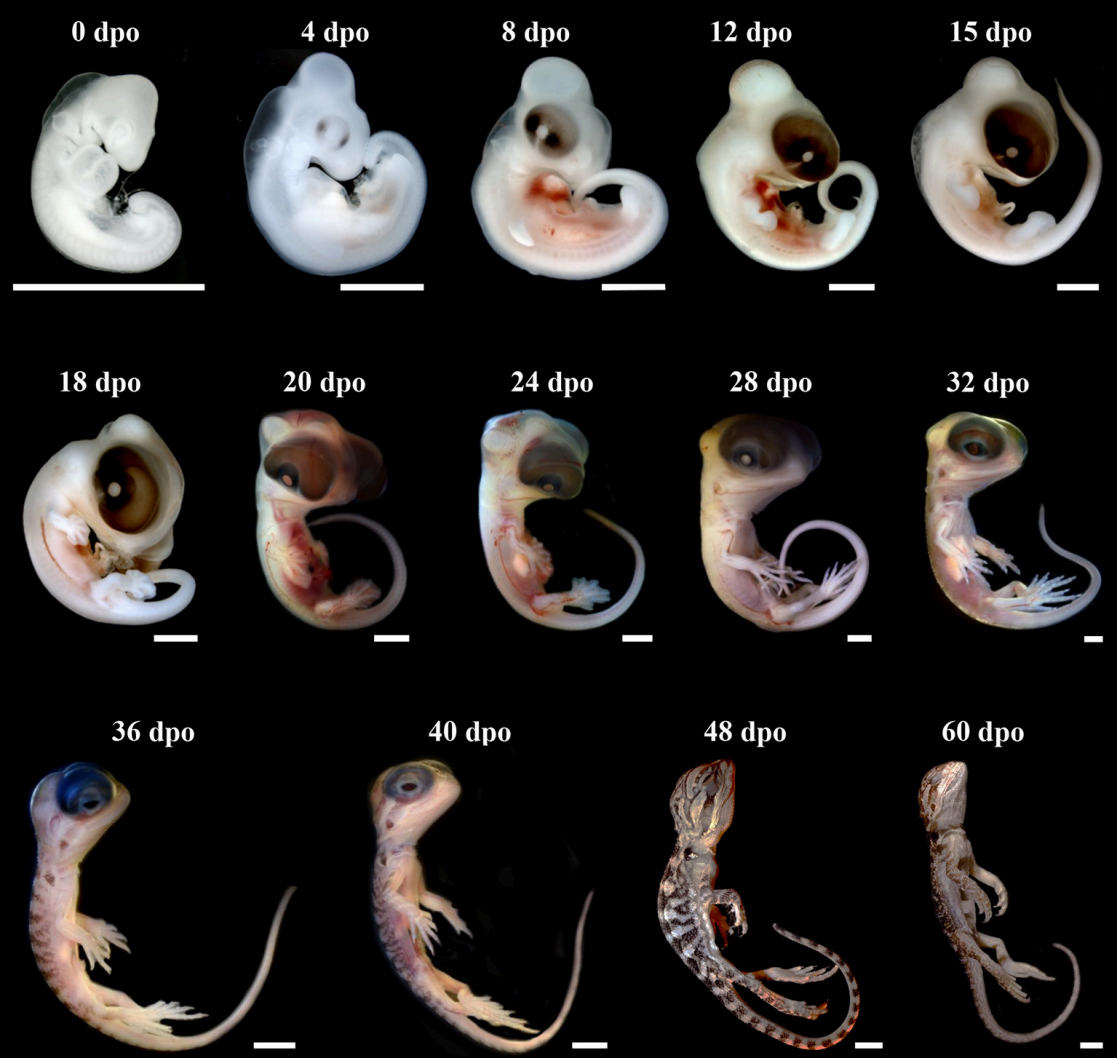
Development of *Pogona vitticeps* embryos. Lateral views of *Pogona vitticeps* embryos at various developmental stages (indicated as embryonic days post-oviposition, dpo) used for SES staging. Scale bars = 1 mm (0–32 dpo) or 5 mm (36–60 dpo).

The external morphology of *P. vitticeps* embryos was examined from oviposition to hatching using the standardized SES staging system (Werneburg, 2009; Additional File 3), which reflects developmental events common to many vertebrates and takes into account heterochronic shifts between species. Additional embryonic characters commonly used in squamates (Dufaure and Hubert, 1961; Sanger et al., 2008; Polachowski and Werneburg, 2013; Werneburg et al., 2015) and/or already described for *P. vitticeps* based on other staging systems (Melville et al., 2016; Whiteley et al., 2017) were also included in our analysis to obtain a unified and complete staging system allowing multi-species comparisons. Initial development, which consists of cleavage, gastrulation, and early organogenesis occurs in the oviduct of *P. vitticeps* before egg laying and could not be assessed. To facilitate staging, different measurements of body and head parameters were also taken in more than 140 embryos (Table 3). We describe 14 embryonic stages covering the whole post-ovipositional period (60 days at 29.5°C) of development of *P. vitticeps* (see Figure 1, Table 3, and complete SES staging in Additional File 3).

**Table 3:** Embryonic staging in *Pogona vitticeps*.

**dpo**	**SES staging**	***Zootoca vivipara* staging table**	***Anolis sagrei* staging table**	**TL (mm)**	**SVL (mm)**
0	1	29	2–3	7	2
4	2	30	4	19	5
8	3	31	5	26	7
12	4	32	6	35	8
16	5	33	7	35	8
18	6	34	8	44	13
20	7	35	9	46	12
24	8	36	10–11	46	12
28	9	37	12–13	48	16
32	10	38	14	55	20
36	11	38	15–16	67	20
40	12	38–39	16–17	71	25
48	12	39	17	100	30
60	13	40	18	128	42

*Stages of embryonic development (indicated as embryonic days post-oviposition, dpo) were estimated based on different embryonic staging tables available for squamates: standard event system SES (Werneburg, 2009), Lacerta vivipara (Dufaure and Hubert, 1961), and Anolis sagrei (Sanger et al., 2008). Relationship between embryonic stage and body size (variance of ±1 mm) is also indicated: TL, total length; SVL, snout-vent length*.

#### SES stage 1 (0 dpo)

**Figure 2:**
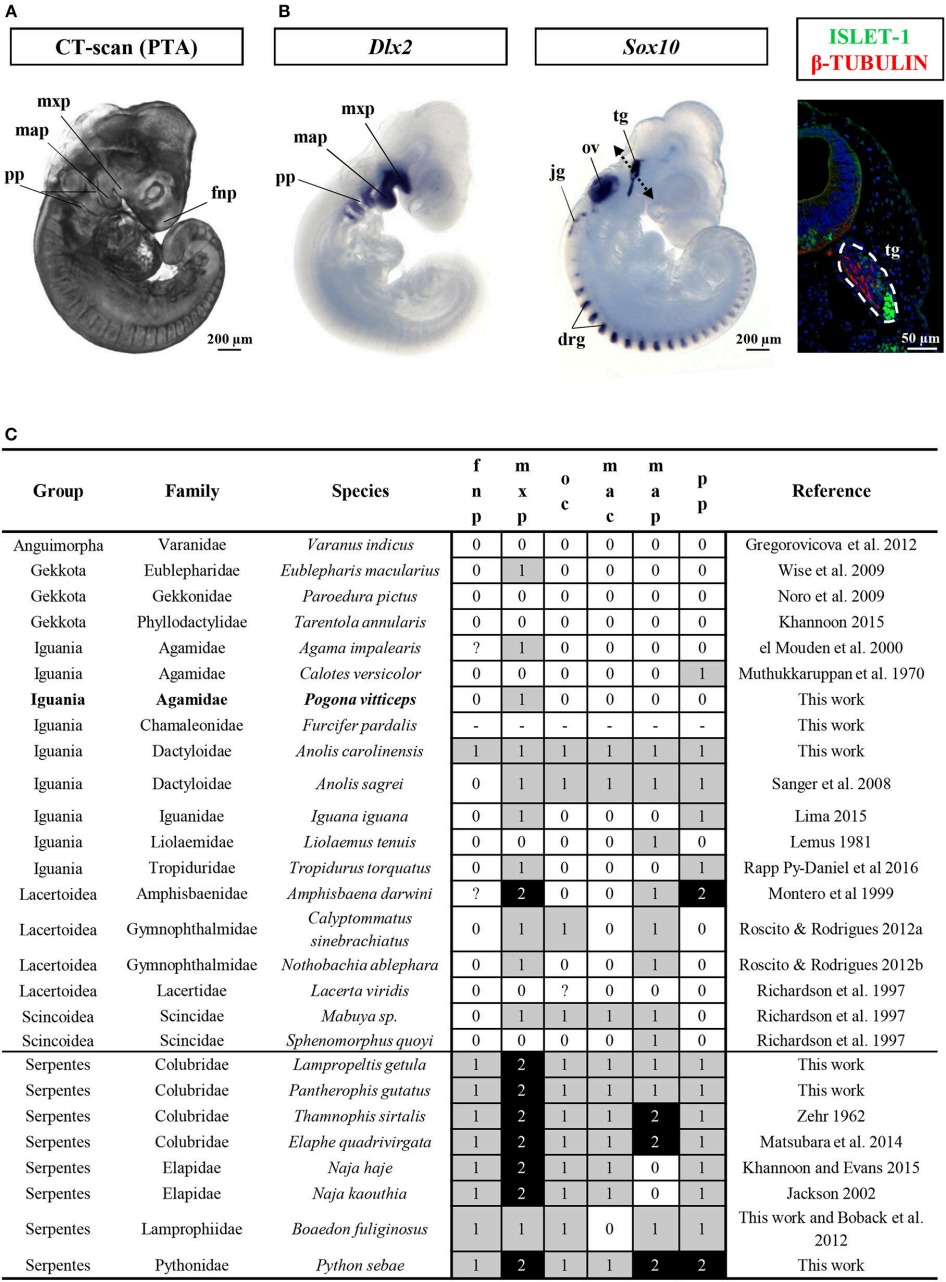
Characterization of craniofacial primordia in squamates. **(A)** Volume rendering image of *Pogona vitticeps* embryo stained with phosphotungstic acid (PTA) at the oviposition stage (0 dpo). **(B)** Whole-mount *in situ* hybridization with cranial neural crest cell markers (*Dlx2*, left panel; *Sox10*, middle panel) or immunohistochemistry with neuronal markers (Islet-1 and β-tubulin, right panel) in *Pogona vitticeps* embryo at 0 dpo. The dashed double-headed arrow in the middle panel indicates the body region processed for sectioning and immunohistochemistry in the right panel. **(C)** Character coding of the degree of development of early craniofacial primordia in lizard and snake embryos at the oviposition stage (0–1 dpo). Species are classified by group and family names. Increasing score number (0–2, see legend in Additional File 9) and grayscale intensity reflect more advanced developmental level of individual traits (fnp, mxp, oc, mac, map, pp). New specimens produced by this work and references for embryos sampled from published literature are indicated. drg, dorsal root ganglia; fnp, frontonasal prominence; jg, jugular ganglion; map, mandibular prominence; mxp, maxillary prominence; oc, oral commissure; ov, otic vesicle; pp, pharyngeal pouch; tg, trigeminal ganglion.

This developmental stage corresponds to Stage 29 of *Z. vivipara* or Stages 2–3 of *A. sagrei*. At this time of oviposition, embryos showed advanced developmental characters such as initial forelimb ridge, anterior cephalic projection, urogenital papilla bud, and more than 30 somites. In addition, the otic vesicle has started to bud from the otic pit (ear region), the optic fissure is present and the contour lens has formed (eye region), and the ventricle assumes its S-shaped form with a well visible ventricle bulbus (heart region). Four pharyngeal arches have already formed, with three pharyngeal slits present, and the maxillary and mandibular prominences are posterior to the whole eye and to the lens region, respectively (Figure 2A and Additional File 3). Interestingly, our broad comparisons of lizard and snake embryos at the oviposition stage demonstrate that such advanced stage of craniofacial primordia is a common but highly variable properties of oviparous squamate species (Figure 2C and Additional File 9) due to the initial retention of developing eggs *in utero* (about one-third of embryogenesis in *P. vitticeps*). Because of our main focus on craniofacial skeleton in this study, we further characterize the developmental stage of cranial neural crest cells (CNCCs)—a migratory population of cells originating from the developing neural tube and giving rise to the majority of the skull bones—, using detection of the expression pattern of specific CNCC markers at oviposition. Two major signals conserved in vertebrates and reflecting different stages of CNCC development (*Sox10*: early stages of CNCC specification and migration; *Dlx2*: CNCC differentiation into ectomesenchyme) were selected (Blentic et al., 2008; Szabo-Rogers et al., 2010; Bhatt et al., 2013). As shown in Figure 2B, *Sox10* is only detected in the otic vesicle, dorsal root ganglia, and developing trigeminal ganglion, thus suggesting the absence of migrating CNCC at the oviposition stage. Coherent with this, *Dlx2*, an ectomesenchymal marker, is strongly expressed in the pharyngeal arches, confirming that the early delamination and migration of CNCCs from the neural tube has already happened and that craniofacial primordia have initiated the condensation of mesenchyme into cartilage and bone (Figure 2B).

#### SES stage 2 (4 dpo)

This developmental stage corresponds to Stage 30 of *Z. vivipara* or Stage 4 of *A. sagrei*. Approximately 40 pairs of somite are visible. External nares have formed. Both the forelimb and hindlimb are at the limb bud stage. Five pharyngeal arches have formed, with four pharyngeal slits still visible, and both the maxillary and mandibular prominences are situated approximately at the midline of the eye. The eye has expanded in size and the lens becomes distinct and faintly pigmented.

#### SES stage 3 (8 dpo)

This developmental stage corresponds to Stage 31 of *Z. vivipara* or Stage 5 of *A. sagrei*. The apical ectodermal ridge (AER) structure is present in both the forelimb and hindlimb. The maxillary prominence has developed rostrally and is located anterior to the lens. Pigmentation of the eye has increased. The cervical flexure remains <90°.

#### SES stage 4 (12 dpo)

This developmental stage corresponds to Stage 32 of *Z. vivipara* or Stage 6 of *A. sagrei*. Both the hindlimb and forelimb are paddle shaped and the forelimb elbow as well as the hindlimb knee are formed. Pharyngeal slits are closed, and the maxillary prominence is anterior to the eye (as the eye size is relatively large in *P. vitticeps*, this position is considered equivalent to being anterior to the eye in other species). The cervical flexure makes a 90° bend. In the darkly pigmented eye, both the pupil and the lower eyelid have started to form.

#### SES stage 5 (15 dpo)

This developmental stage corresponds to Stage 33 of *Z. vivipara* or Stage 7 of *A. sagrei*. The digital plates in both the forelimb and hindlimb are present. The maxillary has fused with the frontonasal prominence. The cervical flexure is now >90°. The skin is still translucent and no scales are evident.

#### SES stage 6 (18 dpo)

This developmental stage corresponds to Stage 34 of *Z. vivipara* or Stage 8 of *A. sagrei*. Digital grooves are visible in the forelimb, while digital serration is present in the hindlimb. The mandibular prominence is anterior to the eye (see also maxillary prominence at *SES Stage 4*). Scleral papillae have started to form in the eye region. Hemipenes become first visible at this stage on either side of the cloaca.

#### SES stage 7 (20 dpo)

This developmental stage corresponds to Stage 35 of *Z. vivipara* or Stage 9 of *A. sagrei*. Rib primordia are clearly visible. The mandibular prominence is at the level of the frontonasal prominence. The cervical flexure has disappeared.

#### SES stage 8 (24 dpo)

This developmental stage corresponds to Stage 36 of *Z. vivipara* or Stages 10–11 of *A. sagrei*. Digits have formed in the hindlimb. The thoracic bulbus has disappeared in the heart region. The mandibular prominence is at the level of the occlusion point. Eyelids have started to overgrow.

#### SES stage 9 (28 dpo)

This developmental stage corresponds to Stage 37 of *Z. vivipara* or Stages 12–13 of *A. sagrei*. The first claws are present in both the forelimb and hindlimb. Scales are visible on the throat, neck, and back only. Eyelids are at the level of scleral papillae. The hemipenes remain external.

#### SES stage 10 (32 dpo)

This developmental stage corresponds to Stage 38 of *Z. vivipara* or Stage 14 of *A. sagrei*. The scleral papillae are inconspicuous. Skin pigmentation is apparent on the back, and ventral scales are visible. The nictitating membrane across the eye is visible.

#### SES stage 11 (36 dpo)

This developmental stage corresponds to Stage 38 of *Z. vivipara* or Stages 15–16 of *A. sagrei*. Eyelids are located ventrally to the lens. Scales are now visible on the eyelid, limb, belly, and tail regions.

#### SES stage 12 (40–48 dpo)

This developmental stage corresponds to Stages 38–39 of *Z. vivipara* or Stages 16–17 of *A. sagrei*. Cranial projections from the developing brain regions have disappeared, and the brain is no longer externally visible. The otic capsule is inconspicuous. Skin pigmentation is spreading over the body and scales fully cover the limb region. Eyelids cover half of the eye. Hemipenes have inverted and are internalized.

#### SES stage 13 (60 dpo)

This developmental stage corresponds to Stage 40 of *Z. vivipara* or Stage 18 of *A. sagrei*. At this time of egg hatchling, the pigmentation and the patterning appear similar as in juveniles and the egg yolk has been fully consumed.

Our comparisons of the developmental sequence of *P. vitticeps* with other lizard staging tables (Table 1) suggest major differences in the developmental timing of some characters, including the late appearance of scales and the relatively delay in the onset of development of eyelids. However, the variable staging criteria and staging tables reported so far in squamate species make direct comparisons extremely difficult. For this reason, we limit here our comparisons to corresponding external characters in a few lizard and snake species with detailed SES staging criteria available [*Z. vivipara*, Lacertidae lizard (Werneburg and Sánchez-Villagra, 2009); *Varanus panoptes*, Varanidae lizard (Werneburg et al., 2015); *Bothropoides jararaca*, Viperidae snake (Polachowski and Werneburg, 2013)], the only standard code reported so far for studying heterochrony and patterns of vertebrate development (Werneburg, 2009). While the overall development of limbs is relatively well-conserved between all lizard species, changes in the developmental sequence of SES characters were observed in *P. vitticeps*: delayed development of the heart (ventricle bulbus) when compared to *Z. vivipara*; delayed but shortened development of the eyelids, prolonged development of the eye, and shortened appearance of the cervical flexure in the neck region when compared to both *Z. vivipara* and *V. panoptes*; delayed apparition and inversion of the hemipenes when compared to *B. jararaca;* and delayed apparition of scales when compared to *B. jararaca* and *Z. vivipara*.

### Shape and bone composition of adult skull

**Figure 3:**
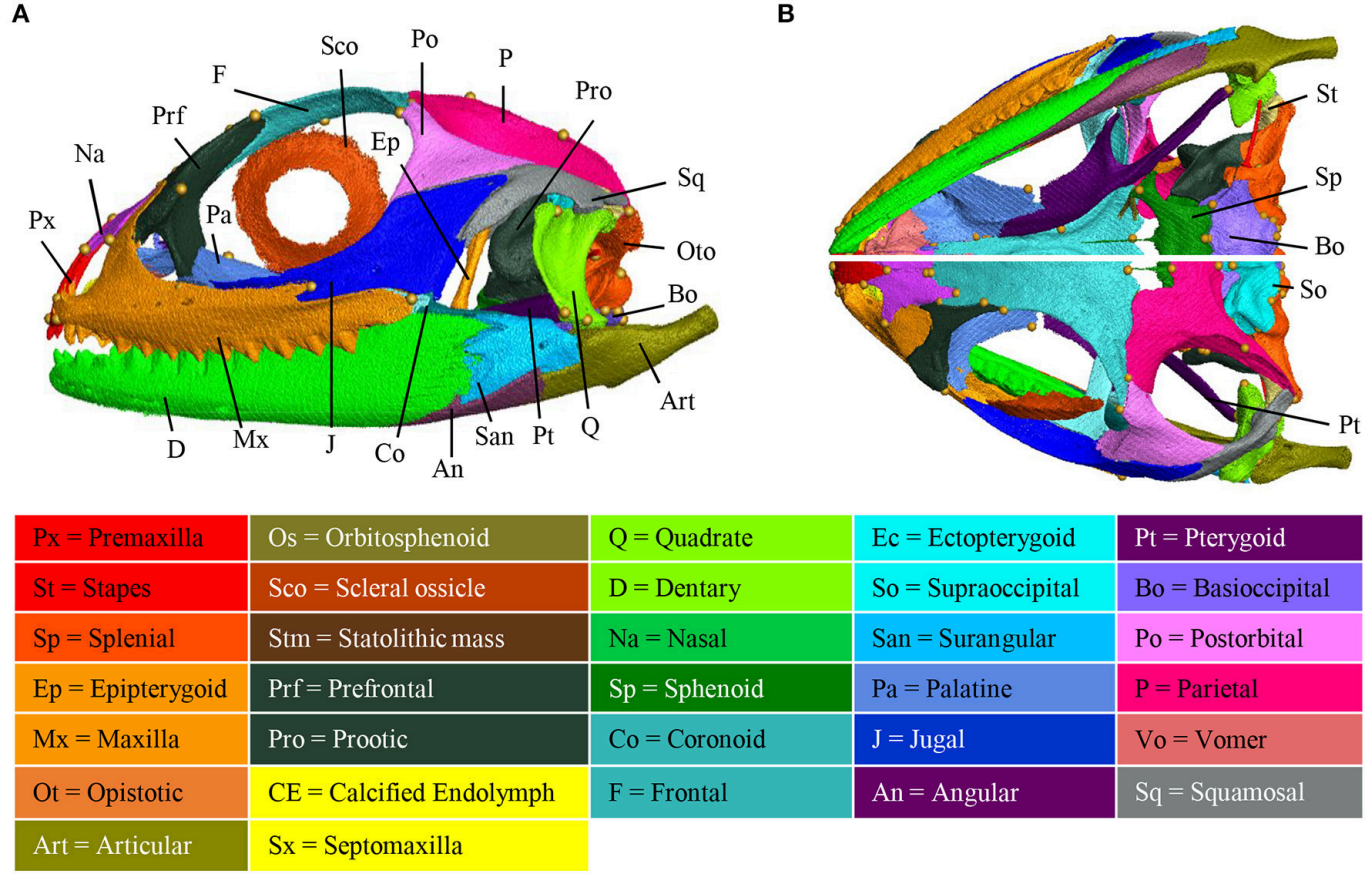
Adult anatomy of the *Pogona vitticeps* skull. Lateral **(A)**, ventral (**B**, top panel), and dorsal (**B**, bottom panel) views of the skull of adult *Pogona vitticeps*. Pictures are based on 3D isosurface renderings and segmentation of individual bones with different colors (see color-coding in the bottom panel).

**Figure 4:**
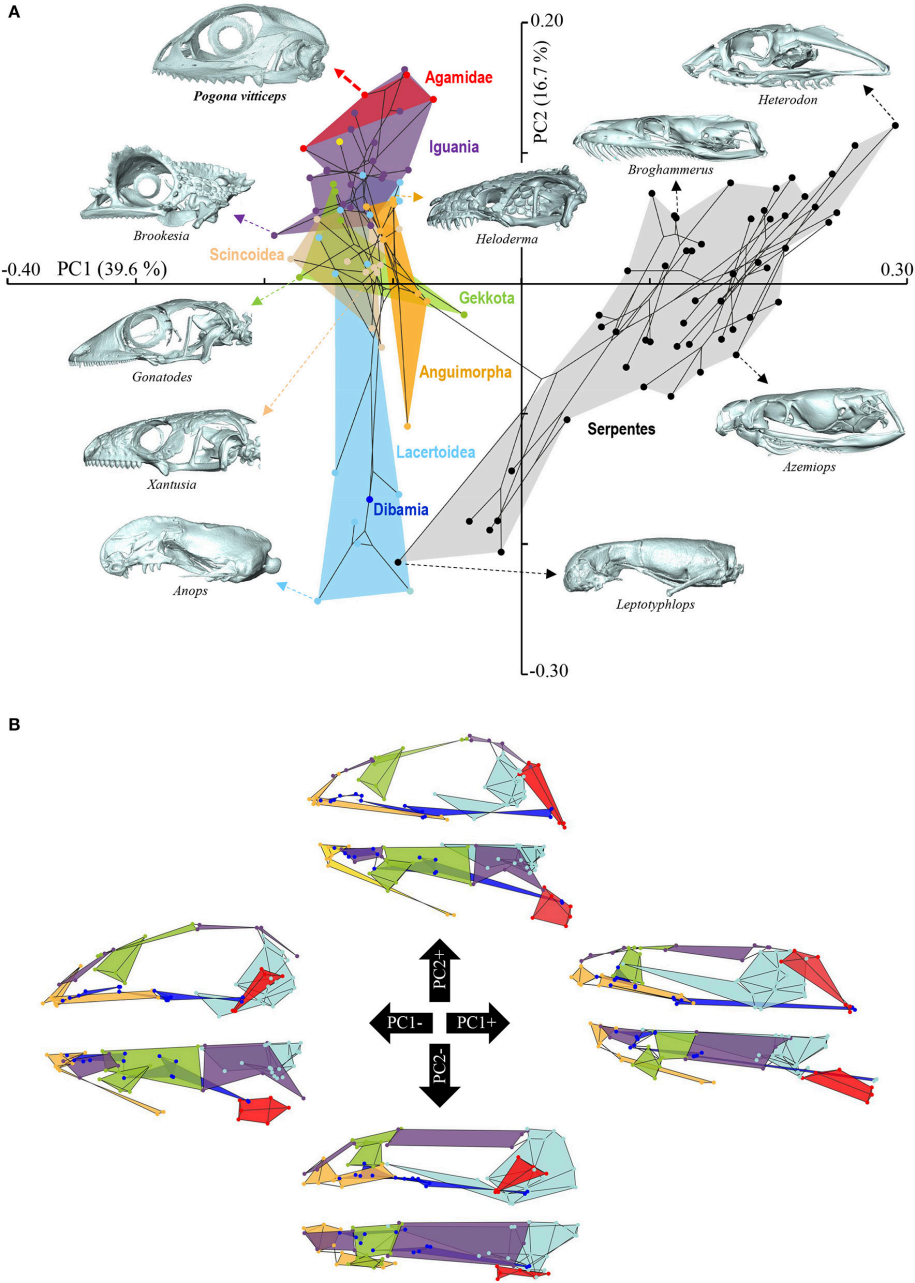
Principal component analysis of cranium shape variation in squamates. **(A)** Phylomorphospace showing the cranium shape distribution of major groups of squamates: Dibamia (dark blue tip node), Gekkota (green shading and tip nodes), Scincoidea (brown shading and tip nodes), Lacertoidea (light blue shading and tip nodes), Anguimorpha (orange shading and tip nodes), Iguania (purple shading and tip nodes), Agamidae (red shading and tip nodes), and Serpentes (gray shading and black tip nodes). Color-codings are as in Additional File 1. Numbers in brackets indicate the percentage of variance explained by each of the PC axes. The 3D rendered craniums (indicated by colored dashed arrows, with species names) corresponding to representative lizard and snake species in both positive and negative directions are shown. The extreme position of *Pogona vitticeps* at positive PC2 values is highlighted in bold. **(B)** For each PC, the extreme cranium shapes at positive (+) and negative (–) values are depicted as wireframe diagrams. Cranium regions showing the greatest variations in shape are indicated by colored landmarks and shadings: quadrate (red), braincase (light blue), palate (dark blue), skull roof (apart from frontal, purple), circumorbital bones (green), tooth-bearing bones (orange). Lateral (top wireframe) and dorsal (bottom wireframe) views of one cranium side are shown for each extreme PC.

**Figure 5:**
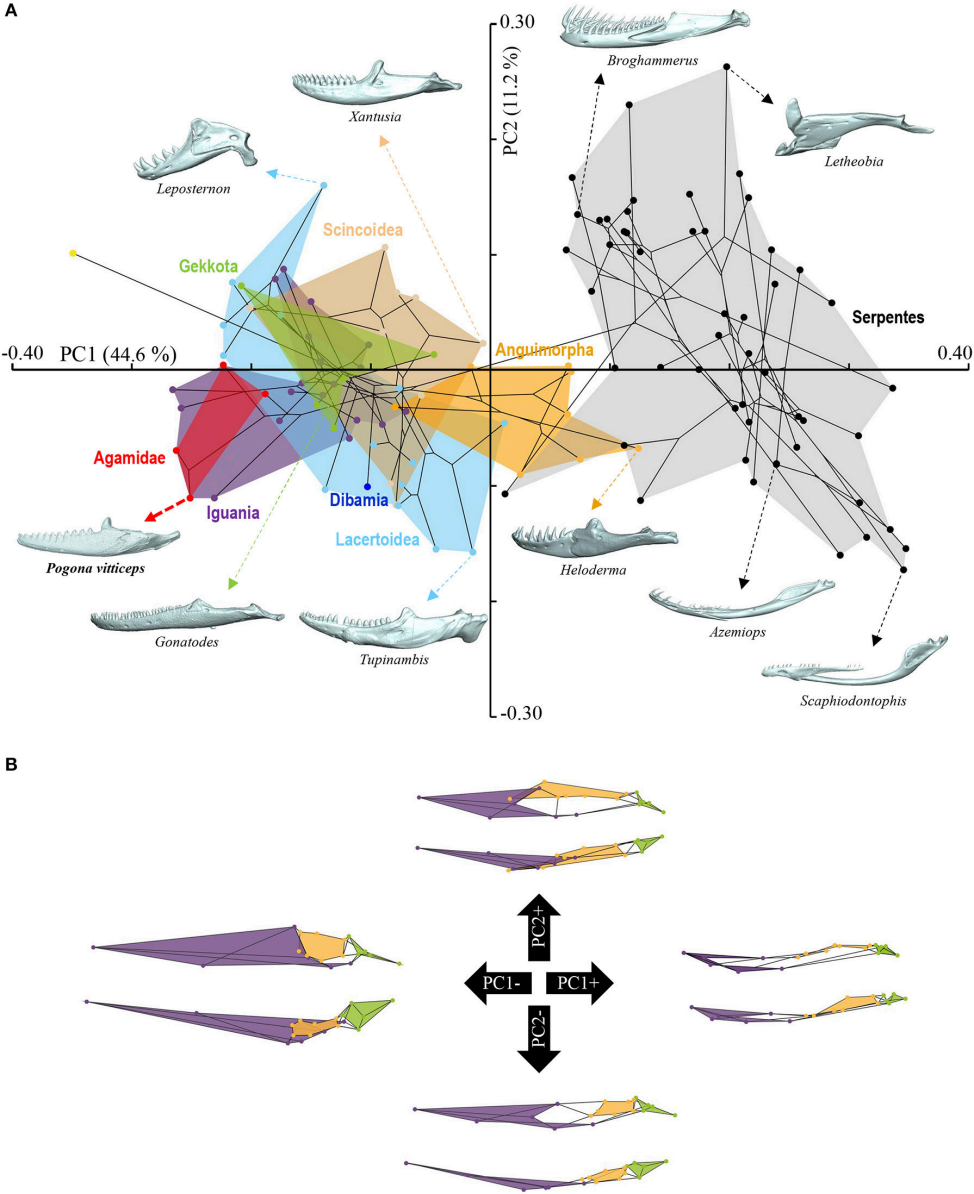
Principal component analysis of mandible shape variation in squamates. **(A)** Phylomorphospace showing the mandible shape distribution of major groups of squamates. Color-codings are as in Figure 4 and Additional File 1. Numbers in brackets indicate the percentage of variance explained by each of the PC axes. The 3D rendered mandibles (indicated by colored dashed arrows, with species names) corresponding to representative lizard and snake species in both positive and negative directions are shown. The extreme position of *Pogona vitticeps* at negative PC1 values is highlighted in bold. **(B)** For each PC, the extreme mandible shapes at positive (+) and negative (–) values are depicted as wireframe diagrams. Mandibular regions showing the greatest variations in shape are indicated by colored landmarks and shadings: dentary and angulo-splenial complex (purple), articular surface and retroarticular process (green), Meckelian fossa (orange). Lateral (top wireframe) and dorsal (bottom wireframe) views of one mandible are shown for each extreme PC.

Several incomplete or complete reports have documented the chondrogenic and/or ossification events in different squamate families, but no agamids have been studied so far (Table 1). To increase this knowledge, we performed a detailed study of the skeletogenesis process in *P. vitticeps*, from onset of ossification to full skull development. We first describe both the general shape and bone composition of the entire skull (cranium and mandible) of adult *P. vitticeps*, based on 3D rendering and segmentation of high-resolution CT-scan data (Figure 3) and 3D geometric morphometric analysis (Figures 4, 5). Our global inspection of CT-scans from adult specimens indicate that the skull of *P. vitticeps* is slightly longer than wide and relatively flattened (Figure 3A). The snout region is robust but relatively compressed in comparison to other parts of the skull. Tooth-bearing elements of the upper jaw form the antero-lateral edges of the upper jaw and stretch form the tip of the snout to the posterior end of the orbit. Orbits lie in the anterior half of the skull and occupy a large proportion of the skull (almost one third of the length and half of the height). The braincase is relatively pronounced and located medially in the posterior part of the skull. The palate lies anteriorly and laterally to the braincase, at the level of its ventral part (Figure 3B). The mandible is predominantly formed by dermatocranial elements and consists of six bones of which dentary forms the major component (Figures 3A,B). To get a more detailed bone composition of the major skull regions in *P. vitticeps*, we next analyzed our accurate segmentation of individual bones from CT-scan data (Figures 3A,B).

#### Circumorbital bones

The orbit is composed of four main dermatocranium bones: the prefrontal anteriorly, the frontal dorsally, the postorbital dorso-posteriorly, and the jugal ventrally, and ventro-posteriorly. While the jugal, prefrontal, and frontal form approximately equal parts of the orbit, the postorbital is relatively smaller in proportion. In addition, both the maxilla and palatine bones contribute to the ventro-anterior part of the orbit.

#### Skull roof

The skull roof is formed by several dermatocranium bones, including the nasals and the parietal anteriorly and posteriorly, respectively, and the frontal medially. The lateral process of the parietal elongates beyond the anterior border of the neurocranium, emphasizing the prominent temporal region. The frontal is the longest skull-roofing bone, thus contributing to the relatively large size of the orbital region.

#### Temporal bones

The temporal region is formed by two dermatocranium bones: the squamosal forms the postero-lateral part, whereas the supratemporal lies attached to the posterior process of the parietal. In addition, the posterior parts of both postorbital and jugal bones contribute to the temporal region. The temporal region is prominent with a large temporal fenestra typical of agamid lizards.

#### Tooth-bearing bones of the upper jaw

The premaxilla and maxillae are dermatocranium, tooth-bearing bones located in the mid-anterior and lateral parts of the upper jaw, respectively. In contrast to the reduced premaxilla, the maxilla is robust and relatively large with bones being connected firmly to the snout, palatal complex, and orbital region.

#### Palatoquadrate derivatives

The epipterygoid is situated above the pterygoid bone, dorso-laterally to the sphenoid, and anteriorly to the prootic. The quadrate forms the articulation between the cranium and mandible, and connects with the articular and the squamosal at both ends. The quadrate is slightly tilted posteriorly and is robust in shape. Both the quadrate and articular bones are part of the splanchnocranium.

#### Palate

The palate is entirely formed by dermatocranium bones: the vomer and palatine form the most anterior part, whereas the long pterygoids stretch posteriorly from the medial part of the upper jaw to the quadrate. The lateral part of the palate is formed by the ectopterygoid that lies between the pterygoid and the posterior part of the maxilla. The small septomaxilla lies between the vomer and the nasal bones but does not connect to any bones.

#### Braincase

The braincase is mainly composed of endochondral bones resulting from both the chondrocranium (braincase) and hyomandibulare (stapes). The floor of the braincase consists anteriorly of the sphenoid, a composite bone of the endochondral basisphenoid and dermatocranial parasphenoid, and posteriorly of the basioccipital. The basioccipital also constitutes the most posterior part of the braincase, forming the ventral edge of the foramen magnum. Two otooccipital bones, formed by the fusion of opisthotic and exoccipital, establish both the lateral edges of the foramen magnum and the posterior part of the fenestra ovalis and otic capsule. The supraoccipital forms the dorsal part of the foramen magnum, otic capsule, and braincase. Prootic bones form the lateral part of the braincase and the anterior part of the fenestra ovalis and otic capsule. The orbitosphenoid consists of two bones that contribute to the lesser wing of the sphenoid bone. Stapes lie laterally to the braincase, with the footplate in the fenestra ovalis.

#### Mandible

The mandible is exclusively composed of dermatocranial bones, apart from the articular proper and retroarticular process. The massive dentary bone constitutes over two thirds of the mandible, stretching from the anterior end of the skull to the level of the neurocranium. The posterior part of the mandible consists of the articular and of three dermatocranium bones: the surangular laterally, the small angular ventrally at the same level as the posterior part of the adductor fossa, and the prearticular. The articular bone results from the fusion of the prearticular (dermatocranium), articular (splanchocranium, Meckelian cartilage), and retroarticular (spanchocranium, part of hyomandibulare). The prearticular part stretches medially to the splenial, whereas the articular surface and retroarticular process form the posteriormost part of the mandible. The coronoid bone is situated in the medial part of the mandible, ventrally to the ectopterygoid and at the same level as the posterior region of the maxilla.

The overall shape of the whole head skeleton of *P. vitticeps* was further extracted (Additional Files 4, 5) and compared with a dataset of adult lizard and snake species covering the majority of squamate lineages (112 species; Additional File 2). Because of the articulation between the cranium and the mandible, these two skull components were treated and analyzed separately. Our 3D morphometric analysis using PCA performed from the Procrustes coordinates of cranium or mandible shape indicates that the two first principal components, PC1 and PC2, provide a good approximation for shape variance as they together account for more than 56% of the total variation in both skull components (Figures 4A, 5A, Additional File 7). The PC1 axis explains the greatest shape variation, representing about 40–45% of the total variance for the cranium and mandible, and clearly separates snakes from lizards which are predominantly located at positive and negative PC1 values, respectively. Analysis of skull shape variations along the PC1 axis indicates substantial changes in all major cranium regions, including the more flattened snout region with angle change of premaxilla, overall reduction of the circumorbital region, anterior expansion of braincase and parietal, different curvature and projection of quadrate, different curvature of vomer, and elongation and narrowing of both palate and pterygoid (Figures 4A,B). Similarly, the mandible show important shape variations along PC1, including the proportional size and curvature of dentary, angle of articular surface and retroarticular process, and size of Meckelian fossa (Figures 5A,B). Additional changes include the level of surangular and prearticular processes and overall anterior-posterior expansion of the mandible. The PC2 axis explains <17% of the total variance for both cranium and mandible, and mainly reflects changes in the cranium component of the skull, including the projection and size of the temporal region, curvature and shape of quadrate, level of anterior expansion of parietal and reduction of frontal, relative size of the circumorbital region compared to the rest of skull, and robustness of maxilla (Figures 4A,B). In the mandibular region, changes along PC2 include the increased size of Meckelian fossa, particularly the surangular region (Figures 5A,B). Interestingly, whereas all lizard cranium morphologies are distributed at negative PC1 values, most agamid species analyzed in our study, including *P. vitticeps* but also *Pogona barbata* and *Agama hispida*, are located among the most extreme positive PC2 values (Figure 4A). Such extreme values confirm the cranium features of *P. vitticeps* (Figure 3) and characterize a triangular skull shape with an enlarged orbital region (linked to the posterior expansion of frontal and maxilla and dorso-medial expansion of prefrontal), a compressed snout with reduced premaxilla, a compressed neurocranium (in particular supraoccipital and cutriform process of basiphenoid), a reduced parietal wall with lateral process of parietal bone elongating beyond the anterior border of the neurocranium, a prominent temporal region, a different projection of the quadrate bone along its antero-posterior axis, as well as an enlarged and robust maxilla with a large prefrontal process. Importantly, our PCA analysis of mandible shape also indicates that agamid species such as *P. vitticeps* and *P. barbata* are located among the most extreme values, in particular along the PC1 axis (Figure 5A). The extreme negative shape of *P. vitticeps* mandible reflects a robust and relatively long structure with enlarged dentary, retroarticular process, and meckelian fossa (Figure 5B). To next assess evolutionary patterns of skull shape among major squamate groups (Gekkota, Scincoidea, Lacertoidea, Anguimorpha, Iguania, and Serpentes), we projected the most-inclusive phylogenetic tree for squamates onto the phenotypic space from the main PC scores (Figures 4A, 5A). As evident from the phylomorphospace plot, a significant phylogenetic signal was observed using multivariate generalized *K*-statistic [*K*-value = 0.63 (cranium) or 0.85 (mandible); *p* = 0.001], and multivariate analysis of variance using MANOVA indicates a significant overall separation of both cranium and mandible shapes among lineages [*F*-value = 6.27 (cranium) or 3.35 (mandible); *p* < 0.007]. Interestingly, *post-hoc* pairwise comparisons further confirmed significant cranium and mandibular shape differences between major squamate lineages, particularly between Serpentes and all lizard groups (Additional File 8). Furthermore, as also indicated by the minimum overlap of Iguania with other groups in patterns of phylomorphospace occupation (Figures 4A, 5A), this lineage significantly diverge from other lizard groups in cranium and/or mandible shape (Additional File 8). Finally, the phylomorphospace approach further highlights the extreme skull shape of agamid species within Iguania (Figures 4A, 5A), but similar comparative analyses with a larger dataset of representative species per families or subfamilies would be needed to confirm skull shape diversification within agamid and/or iguanian lizards.

### Ossification pattern of skull bones during embryogenesis

**Figure 6:**
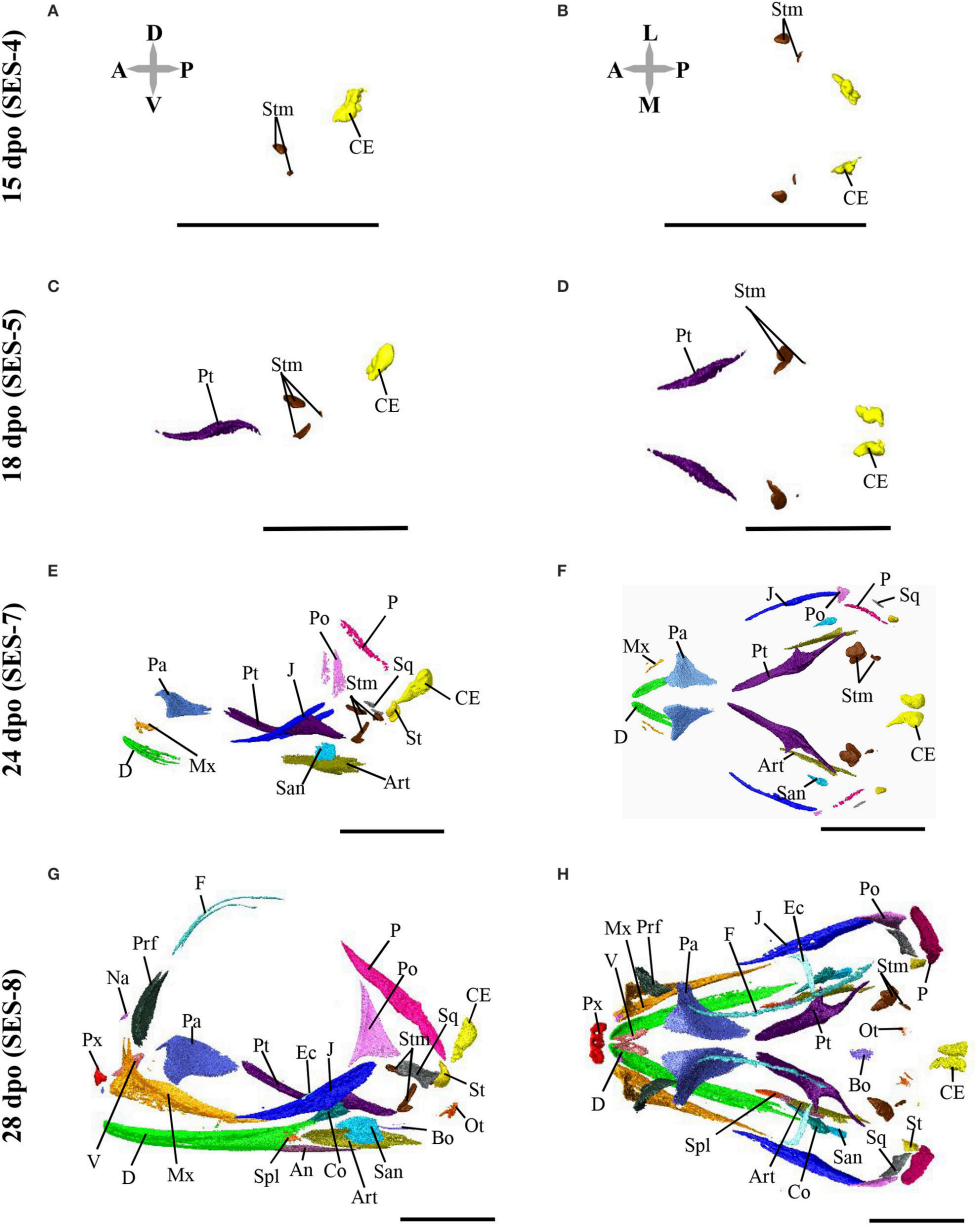
Embryonic development of skull bones in *Pogona vitticeps*, part 1. Lateral **(A,C,E,G)** and dorsal **(B,D,F,H)** views of developing skull in *Pogona vitticeps* embryos at 15 dpo **(A,B)**, 18 dpo **(C,D)**, 24 dpo **(E,F)**, and 28 dpo **(G,H)**. Pictures are based on 3D isosurface renderings and segmentation of individual bones with different colors (see color-coding in Figure 3). Anatomical directions are indicated by gray double-headed arrows: D, Dorsal; P, Posterior; V, Ventral; A, Anterior; L, Lateral; M, Median. Scale bars = 2 mm.

**Figure 7:**
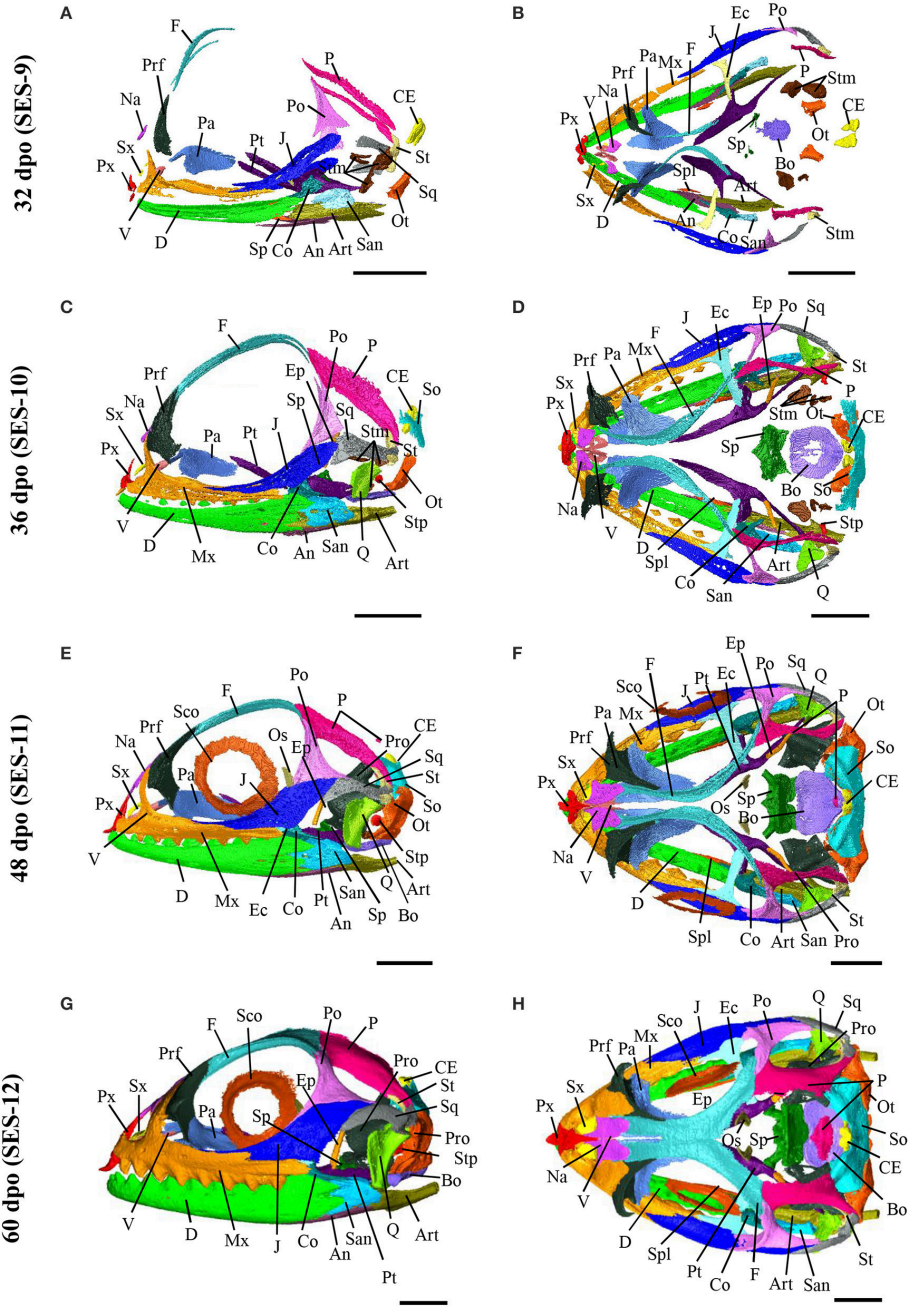
Embryonic development of skull bones in *Pogona vitticeps*, part 2. Lateral **(A,C,E,G)** and dorsal **(B,D,F,H)** views of developing skull in *Pogona vitticeps* embryos at 32 dpo **(A,B)**, 36 dpo **(C,D)**, 48 dpo **(E,F)**, and 60 dpo **(G,H)**. For further details, see Figure 6.

**Figure 8:**
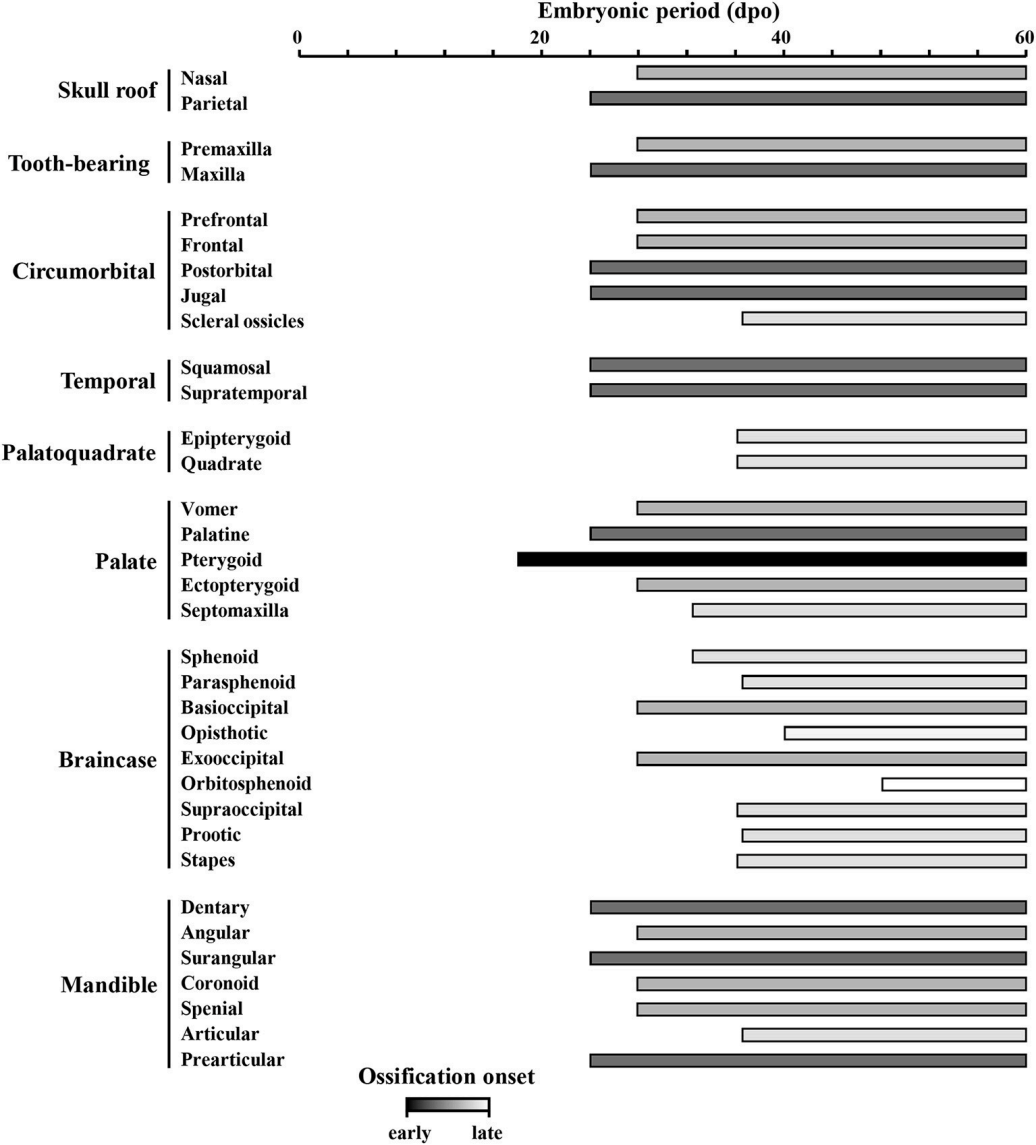
Ossification sequence of skull bones in *Pogona vitticeps*. The presence of ossification in individual skull bones of embryos at particular developmental stages (indicated as embryonic days post-oviposition) is indicated by horizontal bars. Bones are ordered according to main skull regions: skull roof, tooth-bearing bones, circumorbital, temporal, palatoquadrate derivatives, palate, braincase, and mandible. Grayscale intensity reflects the relative timing of onset of ossification, from black (bones ossifying first) to white (bones ossifying last).

Based on the bone composition of the adult *P. vitticeps* skull, we next mapped the ossification pattern (Figures 6, 7) and the sequence of appearance of individual bones (Figure 8) of the entire skull, using high-resolution CT-scan data of developing skulls corresponding to 8 different embryonic stages between 15 and 60 dpo. Our descriptions follow the terminology and major skull regions previously defined for squamates (Evans, 2008):

#### Nasal (skull roof)

The nasals are irregularly-shaped bones that form the upper part of the nasal cavity. Sutures connect the nasals to the premaxilla postero-ventrally and to the prefrontal and frontal antero-dorsally. The anterior part of bones has a hook-like process that overlaps with the facial prominence of the maxillary. Ossification is first visible at 28 dpo as a tiny aggregation between the prefrontals. At 32 dpo, the bone has grown into a flat, triangular shape. Between 36 and 40 dpo, the bone has expanded dorsally and medio-laterally. At 48 dpo, the bone contacts the frontal and has started to expand its processes toward the premaxilla. At 60 dpo, two dorsal processes make contact with both the maxilla and prefrontal. Two nasals fuse together during postnatal development.

#### Parietal (skull roof)

The parietal bones are fused behind the parietal foramen, forming a large and robust quadfurcated bone in the posterior part of the skull roof. The shape resembles the frontal profile of Eurasian lynx on dorsal view. The lateral side forms a large attachment surface for adductor muscles. The post-parietal processes contact the supratemporal dorsally and the squamosum medially, whereas the anterior processes contact the frontal posteriorly and the postorbital medially. Ossification is first visible at 28 dpo as two slender aggregations in the dorso-temporal region. Between 32 and 36 dpo, the ossification expands antero-posteriorly and medially. Between 40 and 48 dpo, the bone contacts the supratemporal and its anterior part expands latero-medially to contact both the frontal and postorbital. At 60 dpo, the lateral part of the ossification have expanded medially and ventrally, forming an attachment surface for muscles on the lateral side. At 48 dpo, a new boomerang-shaped ossification center appears above the neurocranium, antero-dorsally to the calcified endolymph. This medial ossification center has expanded to form a triangular shape by 60 dpo. All the bones fuse during postnatal development.

#### Premaxilla (tooth-bearing bone)

The premaxilla is a slender bone with an irregular shape. It contacts the maxillary anteriorly and the nasal dorso-laterally. Three teeth are visible on the premaxilla. Ossification is first visible at 28 dpo as a trifurcate aggregation with two medial processes and one dorsal process. The tip of the caruncle also starts to ossify at this stage. At 36 dpo, all processes have expanded and the bone becomes quadfurcate by forming an additional pointing hook-like process posteriorly. The caruncle has also ossified toward the premaxilla. Between 40 and 48 dpo, all processes further expand and the bone becomes more robust. At 60 dpo, the premaxilla contacts the nasal and maxillary. In addition, the caruncle is fully developed and has established contact with the premaxilla.

#### Maxilla (tooth-bearing bone)

The maxilla is a large, trifurcate bone with premaxillary, facial, and orbital processes. The premaxillary process is relatively short and contacts the ventral part of the premaxilla medially. The facial processes are wide, flattened, and inflected and contact the dorsolateral part of the nasal and the antero-ventral part of the prefrontal. The orbital process lies mostly under the jugal and contacts the jugal ventrally and the ectopterygoid laterally. The maxilla also contacts the vomer laterally. Four foramina are visible for the maxillary nerve. Ossification is first visible at 28 dpo as a triangular aggregation with both facial and orbital processes in the antero-ventral part of the orbit. At 36 dpo, the premaxillary process has formed and ossification of the reticulum-like parts of the bone has expanded. At 40 dpo, the bone has continued to expand and solidify and now contacts the ectopterygoid. At 48 dpo, the bone is connected to the prefrontal, nasal, and jugal. At 60 dpo, the bone contacts the premaxilla, vomer, and palatine.

#### Prefrontal (circumorbital region)

The prefrontal is a trifurcate bone that lies anteriorly to the orbit and forms a large lacrimal foramen together with the palatine and maxillary. The bone has a robust postero-lateral process and contacts the palate dorsally, the frontal and nasal laterally, and the maxillary posteriorly. Ossification appears at 28 dpo as a curved, leaflet-like aggregation above the maxillary. Between 32 and 36 dpo, the bone expands both ventrally and dorsally and forms a medial ridge toward the facial prominence of the maxillary. At 40 dpo, the postero-lateral process has formed and the bone has reached its adult shape by contacting both the maxillary and palatine. At 48 and 60 dpo, the bone has solidified and contacts the frontal. The bone continues to solidify during postnatal development.

#### Frontal (circumorbital region)

The frontal is a trifurcate bone with narrow anterior widened posterior ends. The anterior part of the bone contacts the nasals postero-dorsally and the prefrontal medially, whereas the posterior part contacts the parietal anteriorly and the postorbitals antero-medially. The parietal foramen is not closed. Ossification is first visible at 28 dpo as two slender curves along the orbit. The latter curves grow and expand alongside the orbit and latero-medially between 28 and 40 dpo. At 48 dpo, the latero-medial expansion has slowly grown, and the bone contacts all nasal, prefrontal, postorbital, and parietal bones. At 60 dpo, two curves start to fuse medially at the midline of the eye.

#### Postorbital (circumorbital region)

The postorbital is a triangular bone situated postero-dorsally to the orbit. It contacts the jugal and squamosum dorso-medially and to the frontal and parietal laterally. The lateral region forms a socket for the aforementioned bones. Ossification is first visible at 28 dpo as a triangular aggregation in the posterior part of the orbit, dorsally to the jugal. Between 32 and 40 dpo, the ossification expands from the tips of the triangle toward the parietal and frontal dorso-medially, the squamosal posteriorly, and the jugal ventrally. It has established contacts with the squamosal, jugal, and parietal at 48 dpo, and with the frontal at 60 dpo. The bone continues to expand during postnatal development.

#### Jugal (circumorbital region)

The jugal is a large bone that contributes to a large portion of the ventral part of the orbit. The dorsal part contacts the squamosal and postorbital medially, whereas the ventral part contacts the maxillary and ectopterygoid dorsally. The ventral part is bifurcate on its anterior side. Ossification is first visible at 24 dpo as a slender curve on the postero-ventral edge of the orbit. Between 28 and 32 dpo, the ossification expands ventrally. At 36 dpo, it has expanded antero-posteriorly alongside the posterior part of the maxilla and toward the postorbital and squamosal. At 40 dpo, the bone contacts the maxilla while several patches continue to ossify. At 48 dpo, the bone contacts the squamosal and postorbital and has reached the adult form.

#### Scleral ossicles (associated with circumorbital region)

The scleral ossicles form horizontal circles in the orbits. The ring consists of 12 plates. Ossification starts at 36 dpo in the dorsal plates. At 40 dpo, the plates have expanded and the ossicles have already reached their adult shape.

#### Squamosal (temporal region)

The squamosal is a robust, curved bone with an irregular shape. Its anterior, bifurcate process contacts both the jugal and postorbital, whereas its posterior part contacts the supratemporal and parietal. Together with the supratemporal, this bone articulates with the quadrate. Ossification is first visible at 24 dpo as a small triangular aggregation in the temporal region. At 28 dpo, the bone has expanded toward the supratemporal and postorbital to become quadrilateral in shape. At 32 dpo, the anterior end is bifurcate and has expanded even closer to the supratemporal. At 36 dpo, this anterior end has expanded toward both the postorbital and jugal and has widened dorso-ventrally. At 40 dpo, the bone has grown anteriorly, while its posterior part has extended dorsally. At 48 dpo, the bone has established contact with the supratemporal, postorbital, and jugal. At 60 dpo, the bone contacts the parietal.

#### Supratemporal (temporal region)

The supratemporal is a curved bone that articulates with the quadrate alongside the squamosal. It contacts the squamosal and the parietal ventro-medially and ventro-laterally, respectively, and forms a socket for the parietal. Ossification is first visible at 24 dpo as a small, droplet-shaped aggregation in the temporal region of the head. At 28 and 32 dpo, the bone has expanded both ventro-medially and dorsally. At 36 dpo, the dorsal part has already developed the posterior socket for the parietal. At 40 dpo, the bone has expanded antero-dorsally alongside the parietal and has developed an articulation surface with the quadrate. At 48 dpo, the bone contacts both the squamosal and parietal and has reached its final adult shape. At 60 dpo, the bone contacts the otooccipital.

#### Epipterygoid (palatoquadrate derivative)

The epipterygoid is a long, slender bone with a horn-shape. The ventral part contacts the dorsal part of the posterior pterygoid via a ball joint. The dorsal parts stretch toward a crest in the lateral border of the parietal. The ventral end lies anteriorly in comparison to the dorsal end. Ossification is first visible at 36 dpo as a tube-like structure lying between the pterygoid and the ossified parietal. At 40 dpo, the bone has expanded both dorsally and ventrally. At 48 dpo, the bone has reached its adult shape and is almost connected to the pterygoid. At 60 dpo, the ball-joint between these bones is formed.

#### Quadrate (palatoquadrate derivative)

The quadrate is a robust, elongated bone with a large ventral surface (showing distinctive condyles) and a well-developed cephalic condyle. The lateral wing of the bone is expanded and contains the tympanic crest. Ossification is first visible at 36 dpo in the future central area of the bone, and parts of the quadrate wing and dorsal condyles have already formed at this stage. At 40 dpo, the bone has expanded anteriorly and posteriorly, and the base of ventral condyles are present. At 48 dpo, the bone has expanded ventrally and reach its adult shape by 60 dpo.

#### Vomer (palate)

The vomers are short, triangular bones lying ventrally to the septomaxilla. Their anterior part shows a short, ventro-medial process. The bones contact the palatine antero-ventrally and form a small contact with the maxillary posteriorly. Ossification is first visible at 28 dpo as ear-shaped aggregations expanding both anteriorly and posteriorly until 36 dpo. At 36 dpo, the two vomer bones have established contact. At 40 dpo, the bones have expanded posteriorly toward the palatine. At 48 dpo, contacts with both the palatine and maxillary have been established, and the bones continue to expand dorsally at 60 dpo and during postnatal development.

#### Palatine (palate)

The palatine is a large bone with three processes: vomerine, maxillary, and pterygoid. The vomerine process is relatively broad with lateral crests in the most anterior part, and contacts the vomer dorsally. The robust maxillary process contacts the prefrontal ventrally and the maxillary medially. The pterygoid process is thin and forms a large connection surface with the pterygoid bone. Ossification is first visible at 24 dpo as a triangular aggregation anteriorly to the pterygoid bone. At 28 dpo, the bone has expanded posteriorly toward both the pterygoid and maxillary. At 32 dpo, the bone has continued to expand antero-ventrally toward the premaxilla. Between 36 and 40 dpo, the bone expands laterally, contacts the pterygoid, and has reached its adult shape. At 48 dpo, the bone has established contact with the vomer, prefrontal, and maxilla. The bone ossification further expands at 60 dpo and during postnatal development.

#### Pterygoid (palate)

The pterygoid is a long, feather-shaped, horizontally lying bone with a posterior quadrate process. It contacts the vomer postero-ventrally and the ectopterygoid ventro-medially. The pterygoid is the first bone to appear in *P. vitticeps* (Figure 8), and ossification is visible as early as 18 dpo as a slender, S-shaped curve in the upper jaw. At 24 dpo, the bone has expanded antero-posteriorly, whereas its posterior part has expanded both dorsally and laterally. At 28 dpo, the bone has grown the surface for the ball joint of the epipterygoid. The lateral process has also expanded toward the ectopterygoid and the anterior and quadrate processes have widened. At 36 dpo, the bone has established contact with the ectopterygoid. At 40 dpo, the lateral process is more robust and the anterior process contacts the palatine. Between 48 and 60 dpo, the bone continues to ossify but has already reached its adult shape.

#### Ectopterygoid (palate)

The ectopterygoid is an irregularly-shaped bone connecting both the medial and lateral parts of the upper jaw. It also contacts the posterior part of the maxillary as well as the jugal and postorbital medially. It articulates laterally with the pterygoid and shows additional process expanding medially into the pterygoid. Ossification is first visible at 28 dpo as an elongated, flat aggregation between the pterygoid and jugal. At 32 dpo, the bone has expanded but retains the same form. At 36 dpo, the medial end of the bone has developed a bifurcate, cup-like end almost connected to the pterygoid. At this stage, the lateral end is triangular, with extensions toward the maxilla anteriorly, the mandible ventrally alongside the jugal, and the dorsal tip of the jugal dorsally. However, the lateral end does not contact any bone yet. At 40 dpo, the bone has further expanded and contacts the pterygoid medially and the maxillary laterally. At 48 dpo, the bone contacts the jugal and has reached its adult shape. At 60 dpo, the lateral end of the ectopterygoid has continued to expand along other bones laterally and it now contacts the postorbital.

#### Septomaxilla (palate)

The septomaxillae are small, concave bones lying horizontally in the nasal cavity and dorsally to the vomer. Their shape resembles a swimming pelagic flatworm. Ossification is first visible at 32 dpo as two separate aggregations lying between the nasal and vomer. At 36 dpo, the aggregations have grown to form a triangular ossification. At 40 dpo, the septomaxilla has further expanded posteriorly to form a ventral curvature and has reached its final shape.

#### Sphenoid (braincase)

The sphenoid is an irregularly shaped bone forming the braincase floor together with the basioccipital. It is formed by the fusion of the endochondral basisphenoid and intramembranous parasphenoid. The basisphenoid is connected with the basioccipital anteriorly and with the prootic ventrally; it also has basipterygoid processes extending toward the pterygoid and forming large condyles. The antero-medial part of the bone is fused with the parasphenoid, which shows a long, thin cutriform process anteriorly as well as a triangular base fused to the basisphenoid. Ossification is first visible at 32 dpo as several fragmented aggregations anteriorly to the forming basioccipital. At 36 dpo, the aggregations have joined together to form a pentagonal shaped ossification (from posterior view) in the antero-ventral part of the neurocranium. At this stage, the parasphenoid has already started to form and fuses with the anterior end of the sphenoid. At 40 and 48 dpo, the basipterygoid processes have started to form, and the sphenoid contacts the basioccipital. At 60 dpo, the bone has expanded and contacts both the prootic and pterygoid. In the juvenile skull, however, the sphenoid-pterygoid connection is still loose, indicating the presence of remaining cartilage between these bones until the full skull size is reached. The cutriform process is not fully ossified at 60 dpo and continues to ossify during postnatal development.

#### Parasphenoid and basisphenoid (braincase)

See sphenoid.

#### Basioccipital (braincase)

In ventral view, the basioccipital appears as a pentagonal bone in the posterior part of the braincase floor. It contacts the sphenoid posteriorly as well as the optoccipital and prootic ventrally. Ossification is first visible at 28 dpo as a small chip-like aggregation lying horizontally in the ventral braincase. At 32 dpo, the bone has expanded to form more anterior ossified structures. At 36 dpo, the bone has formed a disk-like structure with incomplete ossification in its central part. From 40 to 48 dpo, the bone expands from its center to reach the typical hexagonal shape of the adult structure, and now contacts the splenial. At 60 dpo, the bone has further expanded and contacts the otooccipital.

#### Otooccipital (braincase)

The otooccipital is an irregularly shaped bone that resembles a chicken drumstick on lateral view. It is a compound bone that forms from the fusion of the posterior exoccipital and antero-lateral opisthotic bones. The paroccipital process is long and robust, projecting laterally toward contact points with the parietal, squamosal, and supratemporal. The otooccipital forms the lateral and posterior border of the foramen magnum and fenestra ovalis, respectively. The bone contacts the basioccipital dorsally and the prootic and supraoccipital posteriorly. The boundary between the exoccipital and opistotic is not clearly visible in adults, although it can be distinguished during development. Ossification is first visible at 28 dpo as two separate, fragmented aggregations forming the exoccipital part of the bone in the postero-ventral part of the head. At 32 dpo, the exoccipital part has expanded and forms the postero-ventral part of the foramen magnum. At 36 dpo, the exoccipital part continues to expand dorsally and ventro-laterally, and the bone has a mushroom-like shape on dorsal view, resembling that of the adult form. At 40 dpo, the opisthotic has started to form as an irregular thin layer of bone that forms the posterior part of the otic capsule and fuses with the exoccipital. At 48 dpo, the ossification of the opisthotic has expanded anteriorly and the posterior part of the fenestra ovalis has formed. At 60 dpo, the ossification is still in process but the bone has already reached its adult shape and has established contact with the squamosal, supratemporal, parietal, supraoccipital, and exoccipital.

#### Exoccipital and opisthotic (braincase)

See otooccipital.

#### Orbitosphenoid (braincase)

The orbitosphenoid is a curved, boomerang-shaped bone with a widened dorsal end. The bone is vertically oriented, lying ventrally to the frontal and parietal, but does not contact any other bones. It is the last bone to appear in *P. vitticeps* (Figure 8), and ossification is only visible starting from 48 dpo. At this stage, ossification appears as a crescent-shaped aggregation already similar to adult apart from the lack of dorso-lateral processes. Further development of the structure likely happens during postnatal development, as the bone does not differ from 48 to 60 dpo.

#### Supraoccipital (braincase)

The supraoccipital is an irregularly-shaped bone that forms the dorsal parts of the foramen magnum, otic capsule, and sphenoccipital foramen. The bone contacts the otooccipital antero-dorsally and the prootic dorso-medially. The anterior process extends toward the parietal but without establishing direct contact. Ossification is first visible at 36 dpo in the dorsal border of the foramen magnum. At 40 dpo, ossification has spread both laterally and dorsally, and the dorsal parts of the otic capsule have started to form and will continue to ossify until 60 dpo. At 60 dpo, the bone contacts the prootic.

#### Prootic (braincase)

The prootic is an irregular-shaped bone that forms the lateral sides of the braincase and the anterior side of the fenestra ovalis. It shows a prominent foramen for the trigeminal nerve on its ventro-lateral side. It contacts the basioccipital and sphenoid dorsally, the supraoccipital latero-ventrally, and the otooccipital anteriorly. Ossification is first visible at 40 dpo as patchy areas in the future anterior and lateral parts of the bone. At 48 dpo, the bone has ossified posteriorly, forming the anterior border of the fenestra ovalis. At 60 dpo, the ossification is not complete and still proceed during postnatal development.

#### Stapes (associated with braincase)

The stapes form a long, slender, horn-shaped bone lying in the lateral side of the braincase. Its rounded footplate is located in the middle of the fenestra ovalis, stretching laterally to the space behind the quadrate bone. Ossification appears first at 36 dpo as a small tube between the quadrate and neurocranium. At 40 dpo, it has expanded medially and has started to grow the footplate. At 48 dpo, the footplate has reached its adult form. At 60 dpo, the stalk of the bone starts to extend toward the quadrate but the bone continues to develop postnatally.

#### Dentary (mandible)

The dentary is a robust bone with a small bifurcation at its posterior end. The Meckelian groove is open, with margins forming the symphysial surface. The bone contacts the surangular anteriorly, the coronoid laterally, as well as the angular and splenial dorso-laterally. Seven mental foramina were identified on the right side at the anterior end of the bone, whereas only six were found on the left side. The bone starts to ossify at 24 dpo in its anteriormost part. At 28 dpo, it has expanded posteriorly to reach the level of both angular and coronoid bones. It has also formed parts of the symphysical surface. At 36 dpo, the bone has expanded postero-ventrally and contacts the angular. At 40 dpo, the bone has continued to expand both posteriorly and medially, establishing connection with the splenial. At 48 dpo, the bone has expanded postero-medially and contacts the coronoid, articular, and surangular.

#### Angular (mandible)

The angular is a quill-shaped, long, and twisted bone in the postero-ventral part of the mandible. It contacts the surangular and articular bones ventrally and the splenial and dentary bones medially. Ossification is first visible at 28 dpo as a slender aggregation ventral to the mandibular bones. Whereas the bone has expanded at 32 dpo, it only contacts the dentary and articular bones at 36 dpo and the splenial at 40 dpo. At 48 and 60 dpo, the bone has reached the adult form and has established connection with the surangular.

#### Surangular (mandible)

The surangular is a robust bone with a trifurcate anterior part that contacts the dentary postero-medially and forms an intramandibular hinge. The most anterior part of the bone also forms a posterior hinge that prevents the movement of the quadrate. The medial part forms the dorso-medial region of the mandible adductor fossa. It contacts the angular dorsally, the articular dorso-medially, and the coronoid ventrally. Ossification is first visible at 24 dpo as a thick plate laterally to the articular. Between 28 and 32 dpo, it expands anteriorly toward the developing dentary and coronoid. It has established contact with the coronoid and dentary at 36 and 48 dpo, respectively. At 60 dpo, the dorsal part has expanded medially and forms a posterior hinge preventing the sliding of the coronoid.

#### Coronoid (mandible)

The coronoid is a robust, quadfurcate bone with four processes (anterio-medial, dorsal, postero-medial, and posterior). Similarly to other agamids, there is no labial process in *P. vitticeps*. The bone is connected with the dentary ventrally, the splenial dorsally (via antero-medial process), the articular dorso-medially (via both antero- and postero-medial processes), and the surangular dorso-medially (via posterior process). Ossification is first visible at 28 dpo as a sharp needle-like ossification showing the formation of the antero-medial process. At 32 dpo, the ossification has proceeded slowly on the ventral parts of the dorsal process. At 36 and 40 dpo, the bone has reached the adult shape, with all processes being present. At 48 and 60 dpo, the bone contacts the dentary, articular, and surangular bones.

#### Splenial (mandible)

The splenial is a small, triangular bone in the medial part of the mandible. It contacts the angular dorsally, the articular ventrally, and the coronoid ventrally. Ossification is first visible at 28 dpo as a triangular aggregation lying vertically and laterally to the angular and anteriorly to the articular. At 32 and 36 dpo, the bone expands anteriorly. At 40 dpo, the bone shows an anterior process on the dorsal side. At 60 dpo, the bone has expanded posteriorly from its ventral side to reach the adult shape.

#### Articular (mandible)

The articular is fused to the prearticular, resulting in a bone with a wide articular surface, a long anterior prearticular process, a well-developed medial angular process, and a robust retroarticular process pointing dorsally. The prearticular component contacts the splenial postero-medially, the coronoid ventro-laterally, and the angular dorsally. The articular surface lies in the dorsal part of the bone, in contact with the quadrate bone. The articular is also in contact with the surangular medially through both the posterior part of the prearticular process and the antero-lateral part of the articular surface. Ossification is first visible in the prearticular region at 24 dpo toward the middle part of the bone. It appears first as a thin, spearhead shaped ossification in the medial side of the jaw. At 28 and 32 dpo, the prearticular has expanded ventrally, anteriorly, and posteriorly. At 36 dpo, the bone has continued to expand anteriorly to contact both the angular and splenial, and it has fused with the articular proper. The ossification of the articular starts at 36 dpo from its retroarticular process. At 40 and 48 dpo, the ossification has proceeded anteriorly and dorsally, with several small open patches in the bone closed, and the bone has established connections with all dentary, coronoid, and surangular bones. At 60 dpo, the articular surface has ossified and the posterior opening of the retroarticular process (*i.e*. the tip of the process) has closed.

#### Prearticular (mandible)

See articular.

## Discussion

We provide here a complete staging series for the post-oviposition development of the agamid lizard *P. vitticeps*, based on external morphological characters. In particular, we report the first detailed analysis of early craniofacial characters, embryonic skull development, and adult skull shape in this emerging model species. For this purpose, we employed a complementary approach integrating CT-scans, 3D geometric morphometrics and comparative embryology. Such new studies are essential for the establishment and use of well-selected squamate species as Evo-Devo model organisms, particularly for deciphering the developmental mechanisms underlying lineage diversification and phenotypic variation at different taxonomic levels within squamates and vertebrates.

### External features of early craniofacial embryonic development in *Pogona vitticeps*

The majority of cells that form the skeletal and connective tissues of the face are derived from CNCCs that delaminate from the developing neural tube, migrate into facial prominences and pharyngeal arches, and differentiate into ectomesenchyme. Proper patterns of growth and subsequent fusion of the frontonasal, maxillary, and mandibular prominences are especially critical for normal facial formation (for more details on craniofacial development, see, e.g., Blentic et al., 2008; Szabo-Rogers et al., 2010; Bhatt et al., 2013). As a consequence, variation in the timing and relative degree of outgrowth and/or fusion of these prominences could contribute to covariation among facial structures. In *P. vitticeps*, the overall morphological development of craniofacial tissues follows the general trends observed in other squamates. At oviposition, embryos are at Stage 1 (SES) or Stage 29 (*Z. vivipara*) and already display four distinct pharyngeal arches expressing ectomesenchymal markers. Furthermore, both maxillary and mandibular prominences (derived from the first pharyngeal arch) are distinguishable and locate posteriorly to the eye at this stage. Our comparisons with other embryonic species at similar oviposition stage demonstrate that such advanced stage of craniofacial primordia is a common but highly variable properties of squamates. In addition, the inability to access early embryonic stages in *P. vitticeps* indicates that alternative, well-selected models should be preferred to study early skull development aspects such as embryonic origin of bones or precocious CNCC morphogenetic events (delamination, migration). In this context, one of the few squamate species showing pre-ovipositional arrest of early embryonic development such as chameleons (Rafferty and Reina, 2012) could serve as a unique model system.

When compared to other lizard species with detailed SES staging criteria available such as *Z. vivipara* and *V. panoptes* (Werneburg and Sánchez-Villagra, 2009; Werneburg et al., 2015), the overall development of both maxillary and mandibular prominences is relatively more advanced in *P. vitticeps*. In addition, the mandibular process develops relatively slower than the maxillary process, and mostly expands when the maxillary prominence has already developed anteriorly to the eye and has fused with the frontonasal prominence, similarly to the situation in *Z. vivipara* only. Interestingly, and in contrast to other described squamates, the pharyngeal slits fuse during the initial growth of the maxillary prominence in *P. vitticeps*, and not during the mandibular expansion. Altogether, these findings indicate the existence of developmental shifts already during the early patterning of craniofacial primordia in *P. vitticeps*, suggesting that, in addition to skeletal heterochrony previously detected in snakes during early and late ossification events (Werneburg and Sánchez-Villagra, 2015; Da Silva et al., 2018), heterochrony might contribute to divergent skull traits starting from early morphogenetic events in lizards. Importantly, our observations are consistent with the significant albeit reduced facial shape variance previously reported around the pharyngeal arch stage in amniotes using quantitative methods (Young et al., 2014). However, the latter study is only based on a limited number of different embryonic species (especially for squamates), and a further, similar quantitative analysis of ontogenetic trajectories (Alberch et al., 1979) using lizard embryos from multiple species at similar, standardized embryonic stages would be needed to precisely quantify sequence heterochrony and compare shape divergence across lineages and ontogenetic parameters (growth rate, developmental time). Similarly, further comparative investigations of the expression pattern of additional early craniofacial markers and skeletal genes in lizards and snakes might be able to clarify the developmental and genetic mechanisms underlying diversification of squamate skull morphology.

### Skull bone features and ossification patterns

Only few morphological studies have analyzed the evolution of both cranium and mandible morphology across the whole of Squamata. Our new comparative 3D geometric morphometric study reveals new insights into skull evolutionary specializations as well as a remarkable phenotypic diversity among squamate lineages. In particular, major skull shape variations were observed between snakes and lizards in the palatoquadrate, skull roof, and mandibular regions. In addition, a few interesting patterns of convergent evolution in cranium shape, but not mandibular shape, emerge in both snake and legless lizard species with similar fossorial ecological niches. Importantly, as suggested by a previous morphometric survey using a limited number of landmarks on 2D skull data (Stayton, 2005), a more detailed study of the patterns of morphological variation within lizards demonstrates significant skull shape differences between Iguania and Scleroglossa. Coherent with that, the role of sexual dimorphism and adaptation to ecological conditions have already been shown to drive unique skull phenotypic diversity in this group (Melville et al., 2006; Sanger et al., 2013). Among Iguanians, agamid lizards such as *P. vitticeps* show extreme shapes for both cranium and mandibular regions, making them an ideal model system for examining developmental and molecular processes underlying lineage diversification and extreme skull morphologies. The global skull shape of *P. vitticeps*, like that of most agamids, is relatively compressed and triangular. In addition, the shortened snout region and flattened skull are typical morphological specializations of omnivorous lizard species (Metzger and Herrel, 2005), further suggesting relationships between the cranial shape of *P. vitticeps* and ecological factors. Additional bone features common to agamid species include dorsally exposed neurocranium, posteriorly expanded maxilla and dentary reaching the level of the coronoid process, ribbon-like angular, shortened preorbital region, firm ectopterygoid connection to the temporal bones, single reduced premaxilla associated with an antero-medially expanded maxilla, lack of lacrimal bone, and strong palate with firm connections between all dermatocranium bones as well as between palatine and prefrontal. Interestingly, the skull bones of *P. vitticeps* also shares a number of common features with iguanid lizards (as reviewed by Evans, 2008), including lack of postfrontal, large upper temporal fenestra, presence of angular process in the jaw, lack of connection between vomer and premaxilla due to an expanded maxilla, reduced vomer, and expanded rigid palate. The major difference with iguanids resides in fact in the mode of tooth implantation, *P. vitticeps* showing a unique combination of both pleurodont and acrodont teeth in the front and back of their jaws, respectively, rather than only pleurodont teeth. Concomitant with such similarities in skull anatomy, our detailed analysis of the relative timing of onset of ossification in *P. vitticeps* indicates a relatively well-conserved cranial ossification sequence between *P. vitticeps* and other iguanids with literature data available (Werneburg and Sánchez-Villagra, 2015), except for the nasal (dermatocranium), basioccipital (neurocranium), and exooccipital (neurocranium) bones that clearly ossify earlier in *P. vitticeps*. Such overall earlier ossification of endochondral bones within the neurocranium might reflect differences in brain development and encephalization in *P. vitticeps*, as previously shown in mammals (Kobayu et al., 2014). Importantly, our comparison of the agamid *P. vitticeps* with other squamate families confirms the high range of variation in the onset of ossification previously reported among lizards and snakes, thus supporting the important role of heterochrony in the impressive diversification of squamate skull elements (Werneburg and Sánchez-Villagra, 2015; Da Silva et al., 2018). However, conserved cranial patterns were also identified among squamates, including the earlier ossification of the dermatocranium, when compared to both neurocranium and viscerocranium. Especially, the pterygoid is the first bone to appear in *P. vitticeps*, as in many other squamate species, a condition that may be linked to the important role of cranial kinesis in squamates, when compared to other tetrapods. Also, the late appearance of the orbitosphenoid bone is a shared feature of squamates.

In this paper we report the complete post-oviposition development of the agamid lizard *P. vitticeps* based on external morphological characters and ossification patterns, a prerequisite to establishing this organism as a squamate model in Evo-Devo research. In addition, our work indicates that developmental shifts during both early development and ossification of craniofacial tissues have been an important mechanism of evolutionary change in *P. vitticeps*. While these changes might reflect the conserved extreme adult skull shapes and common bone features observed among agamid lizards, further heterochronic studies comparing squamate species with similar number of embryonic and ossification stages, similar definitions of bones (especially composite bones, such as articular, basisphenoid, and exoccipital), and similar detection methods of early craniofacial primordia and ossification pattern should be performed to fully detect evolutionary trends in craniofacial heterochrony. Similarly, the expression patterns and functions of molecular pathways with key roles in early prominence outgrowth and ossification patterning have yet to be elucidated in squamates. Our detailed description of early craniofacial patterning and bone formation in *P. vitticeps* will serve as a valuable basis for future Evo-Devo research investigating the developmental origins of skull variation and diversification at different taxonomic levels.

## Ethics statement

All reptile captive breedings and embryo experiments were approved by the Laboratory Animal Centre (LAC) of the University of Helsinki.

## Author contributions

JO, FD, and ND-P: Designed the experimental approach; JO, KM, and FD: Performed the micro-CT scans; JO and FD: Collected 3D landmark data; JO and FD: Performed all other experiments; JO, FD, and ND-P: Analyzed the data; JO and ND-P: Collected and prepared the *Pogona vitticeps* embryos; JO and ND-P: Prepared the figures and wrote the paper; FD and KM: Contributed in the form of discussion and critical comments. All authors approved the final version of the manuscript.

### Conflict of interest statement

The authors declare that the research was conducted in the absence of any commercial or financial relationships that could be construed as a potential conflict of interest.

## Abbreviations

Evo-Devo: Evolutionary and developmental
CT: micro-computed tomography
SES: Standard Event System
dpo: days post-oviposition
PTA: phosphotungstic acid
SVL: snout-vent length
TL: total length
PFA: paraformaldehyde
PC: principal component
PCA: principal component analysis
WMISH: whole mount *in situ* hybridization
CNCCs: cranial neural crest cells.
