# Supplementary material for: Skull Development, Ossification Pattern, and Adult Shape in the Emerging Lizard Model Organism Pogona vitticeps: A Comparative Analysis With Other Squamates
Source: Front Physiol. 2018 Mar 28;9:278. doi: 10.3389/fphys.2018.00278 (PMC5882870; doi:10.3389/fphys.2018.00278)

Additional file 1. Phylogenetic tree of extant squamate species used in this study, adapted from the most inclusive and recent studies on squamate evolution, and rooted using Sphenodantidae (tuatara). See Additional file 2 for a complete list of species.

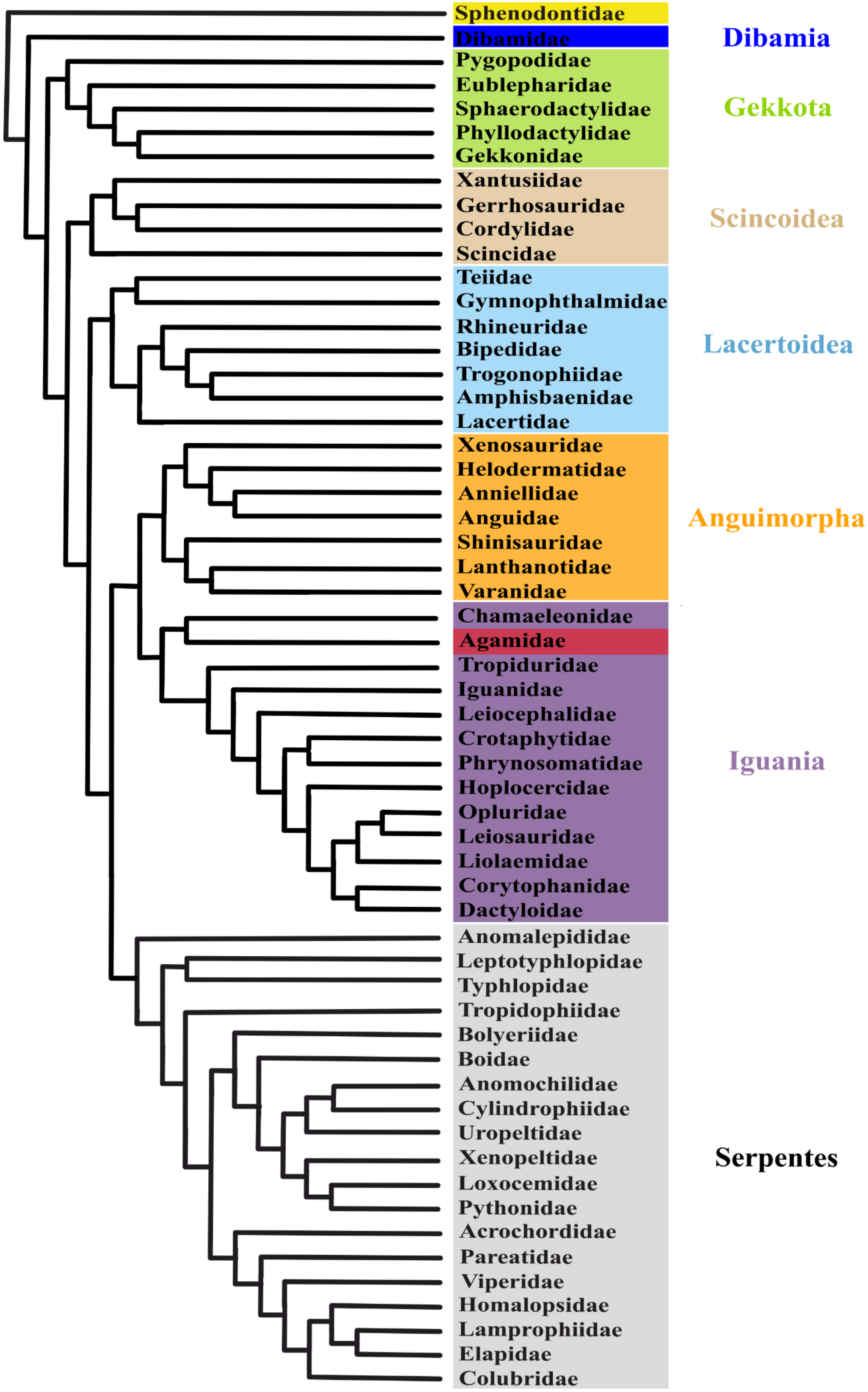

Supplement: Supplementary file 1 [file DataSheet1.PDF]
