# Supplementary material for: Skull Development, Ossification Pattern, and Adult Shape in the Emerging Lizard Model Organism Pogona vitticeps: A Comparative Analysis With Other Squamates
Source: Front Physiol. 2018 Mar 28;9:278. doi: 10.3389/fphys.2018.00278 (PMC5882870; doi:10.3389/fphys.2018.00278)
Supplement: Supplementary file 2 [file DataSheet2.PDF]

Additional file 2. List of identifiers and classifiers for all squamate species used in the study. Species are classified by group and species names.

Groups	Species	References	Families
Lizard	*Aeluroscalabotes felinus*	This work	Eublepharidae
Lizard	*Agama hispida*	Da Silva et al. 2018	Agamidae
Lizard	*Anniella pulchra*	Digimorph	Anniellidae
Lizard	*Anolis carolinensis*	This work	Dactyloidae
Lizard	*Anolis sagrei*	Da Silva et al. 2018	Dactyloidae
Lizard	*Anops kingii*	Digimorph	Amphisbaenidae
Lizard	*Aspidoscelis tigris*	Digimorph	Teiidae
Lizard	*Basiliscus basiliscus*	Digimorph	Corytophanidae
Lizard	*Bipes biporus*	Digimorph	Bipedidae
Lizard	*Brachylophus fasciatus*	Digimorph	Iguanidae
Lizard	*Brachymeles gracilis*	Digimorph	Scincidae
Lizard	*Brookesia brygooi*	Digimorph	Chamaeleonidae
Lizard	*Callopistes maculatus*	Digimorph	Teiidae
Lizard	*Celestus enneagrammus*	Digimorph	Anguidea
Lizard	*Chalarodon madagascariensis*	Digimorph	Opluridae
Lizard	*Chamaesaura anguina*	This work	Cordylidae
Lizard	*Cordylus cordylus*	This work	Cordylidae
Lizard	*Crotaphytus collaris*	Digimorph	Crotaphytidae
Lizard	*Ctenosaura quiquecarinata*	This work	Iguanidae
Lizard	*Dibamus novaeguineae*	Da Silva et al. 2018	Dibamidae
Lizard	*Diplometopon zarudnyi*	Digimorph	Trogonophiidae
Lizard	*Egarnia whitii*	This work	Scincidae
Lizard	*Enyalioides laticeps*	Digimorph	Hoplocercidae
Lizard	*Eulamprus quoyii*	Da Silva et al. 2018	Scincidae
Lizard	*Gambelia wislizenii*	Digimorph	Crotaphytidae
Lizard	*Gekko gecko*	This work	Gekkonidae
Lizard	*Gerrhonotus infernalis*	Da Silva et al. 2018	Anguidae
Lizard	*Gerrhosaurus skoogi*	Digimorph	Gerrhosauridae
Lizard	*Gonatodes albogularis*	Digimorph	Sphaerodactylidae
Lizard	*Heloderma suspectum*	Digimorph	Helodermatidae
Lizard	*Iguana iguana*	This work	Iguanidae
Lizard	*Kentropyx altamazonica*	Da Silva et al. 2018	Teiidae
Lizard	*Lanthanotus borneensis*	Digimorph	Lanthanotidae
Lizard	*Leiocephalus barahonensis*	Digimorph	Leiocephalidae
Lizard	*Leiocephalus schreibersii*	This work	Leiocephalidae
Lizard	*Lepidophyma smithii*	Digimorph	Xantusiidae
Lizard	*Leposternon microcephalum*	Digimorph	Amphisbaenidae
Lizard	*Lialis burtonis*	Digimorph	Pygopodidae

Lizard	*Pholidobolus montium*	Digimorph	Gymnophthalmidae
Lizard	*Phymaturus palluma*	Digimorph	Liolaemidae
Lizard	*Pogona barbata*	Da Silva et al. 2018	Agamidae
Lizard	*Pogona vitticeps*	This work	Agamidae
Lizard	*Pristidactylus torquatus*	Digimorph	Leiosauridae
Lizard	*Rhineura floridana*	Digimorph	Rhineuridae
Lizard	*Shinisaurus crocodilurus*	Digimorph	Shinisauridae
Lizard	*Takydromus sexlineatus*	This work	Lacertidae
Lizard	*Tarentola mauritanica*	Da Silva et al. 2018	Phyllodactylidae
Lizard	*Teius teyou*	This work	Teiidae
Lizard	*Tiliqua scincoides*	Da Silva et al. 2018	Scincidae
Lizard	*Trogonophis wiegmanni*	Digimorph	Trogonophiidae
Lizard	*Tupinambis teguixin*	Digimorph	Teiidae
Lizard	*Uma scoparia*	Digimorph	Phrynosomatidae
Lizard	*Uranoscodon superciliosus*	Digimorph	Tropiduridae
Lizard	*Uromastyx hardwickii*	Digimorph	Agamidae
Lizard	*Uta stansburiana*	Digimorph	Phrynosomatidae
Lizard	*Varanus acanthurus*	Digimorph	Varanidae
Lizard	*Xantusia riversiana*	Digimorph	Xantusiidae
Lizard	*Xenosaurus grandis*	Digimorph	Xenosauridae
Lizard	*Zootoca vivipara*	Da Silva et al. 2018	Lacertidae
Snake	*Acanthophis antarcticus*	Da Silva et al. 2018	Elapidae
Snake	*Acrochordus granulatus*	Da Silva et al. 2018	Acrochordidae
Snake	*Amblyodipsas unicolor*	Da Silva et al. 2018	Lamprophiidae
Snake	*Anomochilus leonardi*	Digimorph	Anomochilidae
Snake	*Aparallactus modestus*	Da Silva et al. 2018	Lamprophiidae
Snake	*Aplopeltura boa*	Da Silva et al. 2018	Pareatidae
Snake	*Arrhyton taeniatum*	Da Silva et al. 2018	Colubridae
Snake	*Aspidites melanocephalus*	Digimorph	Pythonidae
Snake	*Atractaspis boulengeri*	Da Silva et al. 2018	Lamprophiidae
Snake	*Azemiops kharini*	Da Silva et al. 2018	Viperidae
Snake	*Bitis arietans*	Da Silva et al. 2018	Viperidae
Snake	*Boa constrictor*	Da Silva et al. 2018	Boidae
Snake	*Boaedon fuliginosus*	Da Silva et al. 2018	Lamprophiidae
Snake	*Bothrops jararacussu*	Da Silva et al. 2018	Viperidae
Snake	*Calabaria reinhardtii*	Digimorph	Boidae
Snake	*Candoia superciliosa*	Da Silva et al. 2018	Boidae
Snake	*Corallus hortulanus*	Da Silva et al. 2018	Boidae
Snake	*Casarea dussumieri*	Digimorph	Bolyeriidae
Snake	*Coronella austriaca*	Da Silva et al. 2018	Colubridae
Snake	*Cylindrophis melanotus*	Da Silva et al. 2018	Cylindrophiidae
Snake	*Daboia russelii*	Da Silva et al. 2018	Viperidae
Snake	*Dasypeltis scabra*	Da Silva et al. 2018	Colubridae
Snake	*Duberria lutrix*	Da Silva et al. 2018	Lamprophiidae
Snake	*Eirenis decemlineatus*	Da Silva et al. 2018	Colubridae
Snake	*Eirenis rothii*	Da Silva et al. 2018	Colubridae
Snake	*Eryx jaculus*	Da Silva et al. 2018	Boidae
Snake	*Heterodon platirhinos*	Da Silva et al. 2018	Colubridae
Snake	*Hydrophis gracilis*	This work	Elapidae
Snake	*Lampropeltis getula*	Digimorph	Colubridae
Snake	*Leptotyphlops dulcis*	Digimorph	Leptotyphlopidae

Snake	*Letheobia caeca*	This work	Typhlopidae
Snake	*Liotyphlops albirostris*	Digimorph	Anomalepididae
Snake	*Loxocemus bicolor*	Digimorph	Loxocemidae
Snake	*Lycodon aulicus*	Da Silva et al. 2018	Colubridae
Snake	*Natrix natrix*	Da Silva et al. 2018	Colubridae
Snake	*Nerodia sipedon*	Da Silva et al. 2018	Colubridae
Snake	*Opisthotropis latouchii*	Da Silva et al. 2018	Colubridae
Snake	*Pantherophis obsoletus*	Da Silva et al. 2018	Colubridae
Snake	*Pareas carinatus*	Da Silva et al. 2018	Pareatidae
Snake	*Polemon gabonensis*	Da Silva et al. 2018	Lamprophiidae
Snake	*Prosymna ambigua*	Da Silva et al. 2018	Lamprophiidae
Snake	*Psammophis sibilans*	Da Silva et al. 2018	Lamprophiidae
Snake	*Pseudechis porphyriacus*	Da Silva et al. 2018	Elapidae
Snake	*Python bivittatus*	Da Silva et al. 2018	Pythonidae
Snake	*Python reticulatus*	This work	Pythonidae
Snake	*Scaphiodontophis annulatus*	Da Silva et al. 2018	Colubridae
Snake	*Sibynophis collaris*	Da Silva et al. 2018	Colubridae
Snake	*Tropidophis haetianus*	Digimorph	Tropidophiidae
Snake	*Typhlops richardi*	Da Silva et al. 2018	Typhlopidae
Snake	*Ungaliophis continentalis*	Digimorph	Boidae
Snake	*Uropeltis continentalis*	Digimorph	Uropeltidae
Snake	*Xenopeltis unicolor*	Digimorph	Xenopeltidae
Tuatara	*Sphenodon punctatus*	Digimorph	Sphenodontidae
