# Supplementary material for: Skull Development, Ossification Pattern, and Adult Shape in the Emerging Lizard Model Organism Pogona vitticeps: A Comparative Analysis With Other Squamates
Source: Front Physiol. 2018 Mar 28;9:278. doi: 10.3389/fphys.2018.00278 (PMC5882870; doi:10.3389/fphys.2018.00278)
Supplement: Supplementary file 3 [file DataSheet3.PDF]

Additional file 3. SES staging table for *Pogona vitticeps* embryos based on Wernerburg 2009.

Standard Event System for Vertebrate Embryology

species (group)	stage/specimen	Specimen	specimen/stage No.	
Pogona vitticeps	breeding temp.	29.5	collection No.	
	age (days)	E0-60	sheet No.	1 /

CC	SEC	SE	↓
egg	V01a	egg lay	E0
blastula	V02a	blastoporus	
neural tube	V03a	primitive streak	
	V03b	neural folds closure	
	V03c	anterior neuropore closed	E0
	V03d	posterior neuropore closed	E0
somites	V04a	somites hard count	E8
	V04b	1-5 somite pairs	
	V04c	6-10 somite pairs	
	V04d	11-15 somite pairs	
	V04e	16-20 somite pairs	
	V04f	21-25 somite pairs	
	V04g	26-30 somite pairs	
	V04h	31-35 somite pairs	E0
	V04i	36-40 somite pairs	E4
	V04j	41-45 somite pairs	
	V04k	46-50 somite pairs	
	V04l	51-55 somite pairs	
	V04m	56-60 somite pairs	
	V04n	61 and more somite pairs	
head	V05a	head bulbus	
	V05b	anterior cephalic projection	E0
	V05c	head projection disappeared	E40
nose	V06a	olfactory pit	E0
	V06b	external nares	E4
ear	V07a	otic pit	
	V07b	otic vesicle	E0
	V07c	otic capsule inconspicuous	E40
eye	V08a	optic vesicle	
	V08b	lens vesicle	
	V08c	optic fissure	E0
	V08d	contour lens/iris	E0
	V08e	pupil forms	E18
	V08f	scleral papillae	E18
	V08g	scleral papillae inconspicuous	E32
ribs	V09a	rib primordia	E20
heart	V10a	Ventricle bulbus	E0
	V10b	thoracal bulbus disappeared	E24
	V10c	ventricle S-shaped	E0
tail	V11a	tail bud	
limbs	V12a	forelimb ridge	E0
	V12b	forelimb bud	E4
	V12p	hindlimb bud	E4
	V12c	forelimb elongated	
	V12p	hinlimb elongated	
	V12d	forelimb AER	E8
	V12e	hindlimb AER	E8
	V12f	forelimb elbow	E12
	V12t	hindlinb knee	E12
	V12g	forelimb paddle	E12
	V12h	hindlimb paddle	E12
	V12i	forelimb digital plate	E15
	V12j	hindlimb digital plate	E15
	V12k	digital grooves (forelinb)	E18
	V12l	digital serration (hindlimb)	E18
	V12m	finger	E24
	V12w	toe	
	V12n	first claw (forelimb)	E28
	V12x	first claw (hindlimb)	E28

CC	SEC	SE	↓
scales/etc.	V13a	head scales	E36
	V13b	throat scales	E28
	V13c	eyelid scales	E36
	V13d	neck scales	E28
	V13e	back scales	E28
	V13f	limb scales	E36
	V13g	whole forelimb scales	E40
	V13h	tail scales	E36
	V13i	carapace scutes	
hatch	V14a	hatch	E60
maxillary process	G01a	max bud	
	G01b	max posterior eye	E0
	G01c	max midline eye	E4
	G01d	max anterior lens	E8
	G01e	max anterior eye	E12
	G01f	max frontonasal fuse	E15
mandibular process	G02a	mand arch bud	
	G02b	mand posterior eye	
	G02c	mand posterior lens	E0
	G02d	mand midline eye	E4
	G02e	mand anterior lens	E15
	G02f	mand anterior eye	E18
	G02g	mand level frontonasal	E20
	G02g	mand occlusion point	E24
pharyngeal arches	G03a	2nd arch	E0
	G03b	3rd arch	E0
	G03c	4th arch	E0
	G03d	5th arch	E4
	G03e	hyoid flap	
pharyngeal slits	G04a	1st slit	E0
	G04b	2nd slit	E0
	G04c	3rd slit	E0
	G04d	4th slit	E4
	G04e	slits closed	E12
urogenital papillae	G05a	urogenital papilla bud	E0
	G05b	urogenital papilla inconspicuous	E40
neck	T01a	cervical flexure 90°	E12
	T01b	cervical flexure disappeared	E20
	T01c	wrinkles on neck	E24
eye lids	A01a	lower lid	E12
	A01b	eyelid begun overgrow	E24
	A01c	eyelid at scleral papillae	E28
	A01d	eyelid ventral lens	E36
	A01e	eyelid half eye	E40
	A01f	membrana nictitans	E32
	A02a	caruncle	
caruncle	Sq01a	Hemipenis visible	E18
Hemipenis	Sq01b	Hemipenes inverted	E40

Standard Event System for Vertebrate Embryology

species (group)	stage/specimen	Specimen	specimen/stage No.	PV1
Pogona vitticeps	breeding temp.	29.5	collection No.	
	age (days)	0 dpo	sheet No.	2 / 11

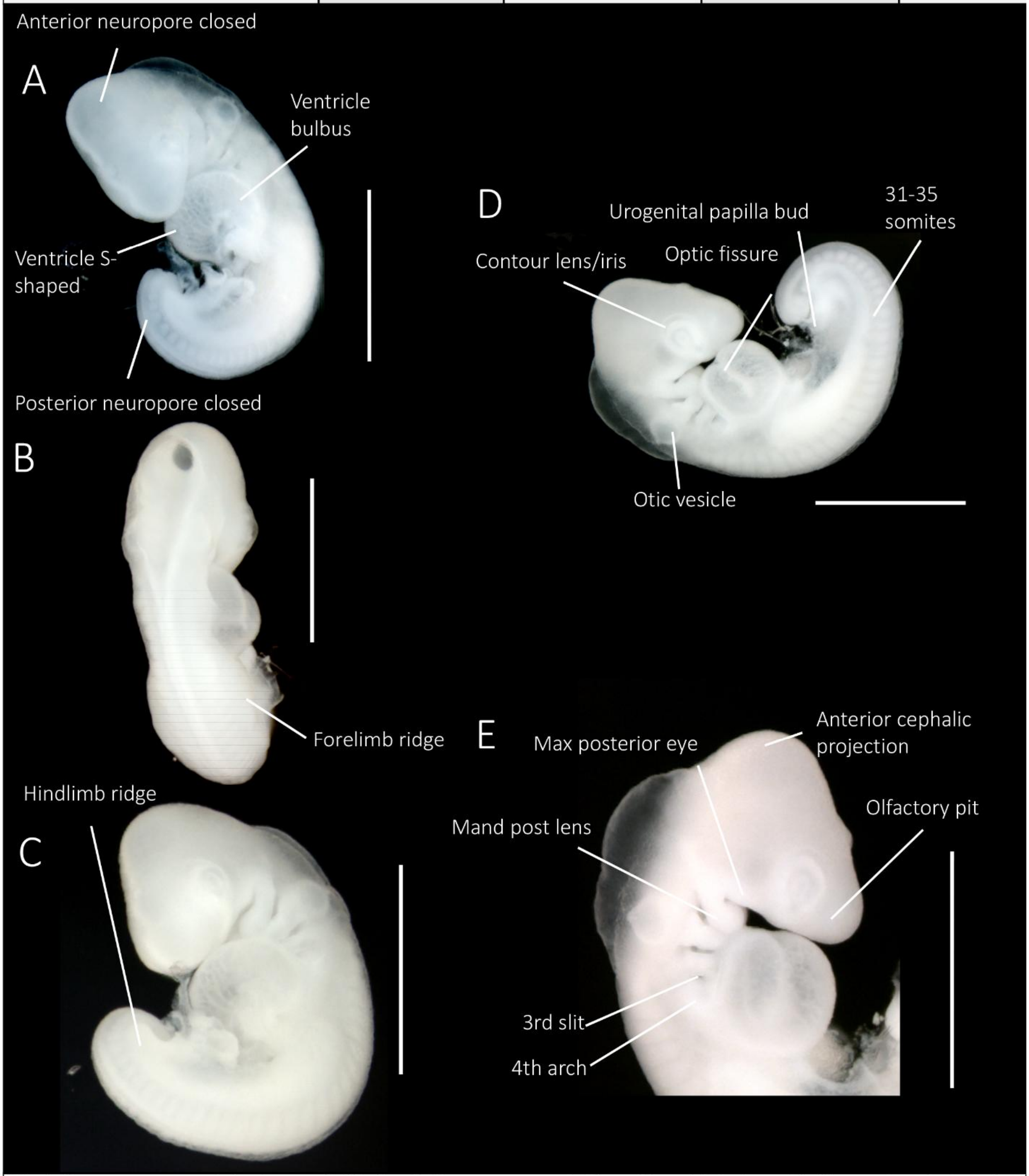

Fig. 1: *Pogona vitticeps* at 0 dpo in ventral (A), dorsal (B), postero-ventral (C), and lateral (D) views. (E) Close-up lateral view of cranial and pharyngeal regions. Scale bars = 1 mm.

Standard Event System for Vertebrate Embryology

species (group)	stage/specimen	Specimen	specimen/stage No.	PV2
Pogona vitticeps	breeding temp.	29.5	collection No.	
	age (days)	4 dpo	sheet No.	3 / 11

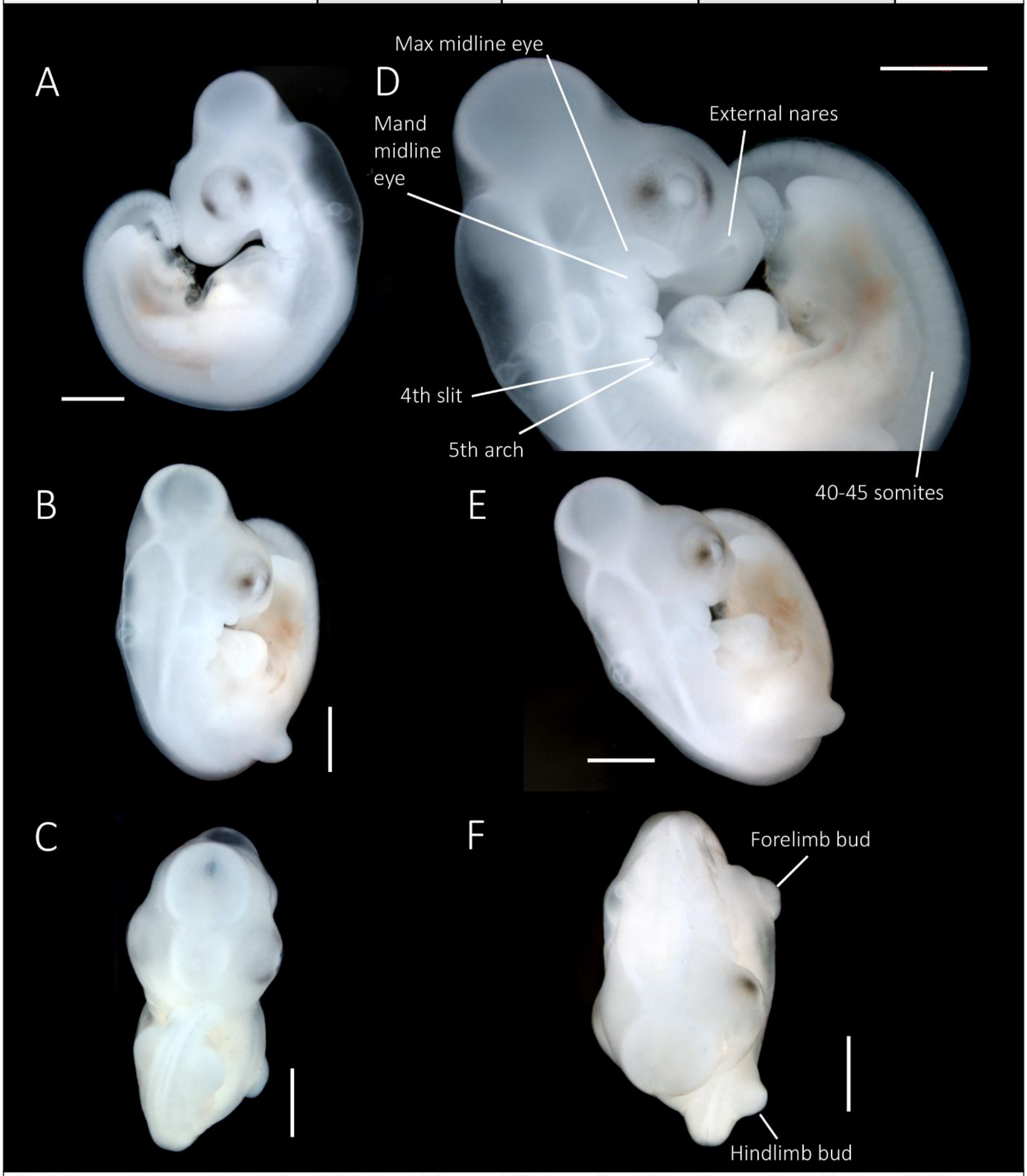

Fig. 2: *Pogona vitticeps* at 4 dpo in lateral (A), dorsal (B), ventral (C), latero-dorsal (E), and antero-dorsal (F) views. (D) Close-up latero-dorsal view. Scale bars = 1 mm.

species (group)	stage/specimen	Specimen	specimen/stage No.	PV3
Pogona vitticeps	breeding temp.	29.5	collection No.	
	age (days)	8 dpo	sheet No.	4 / 11

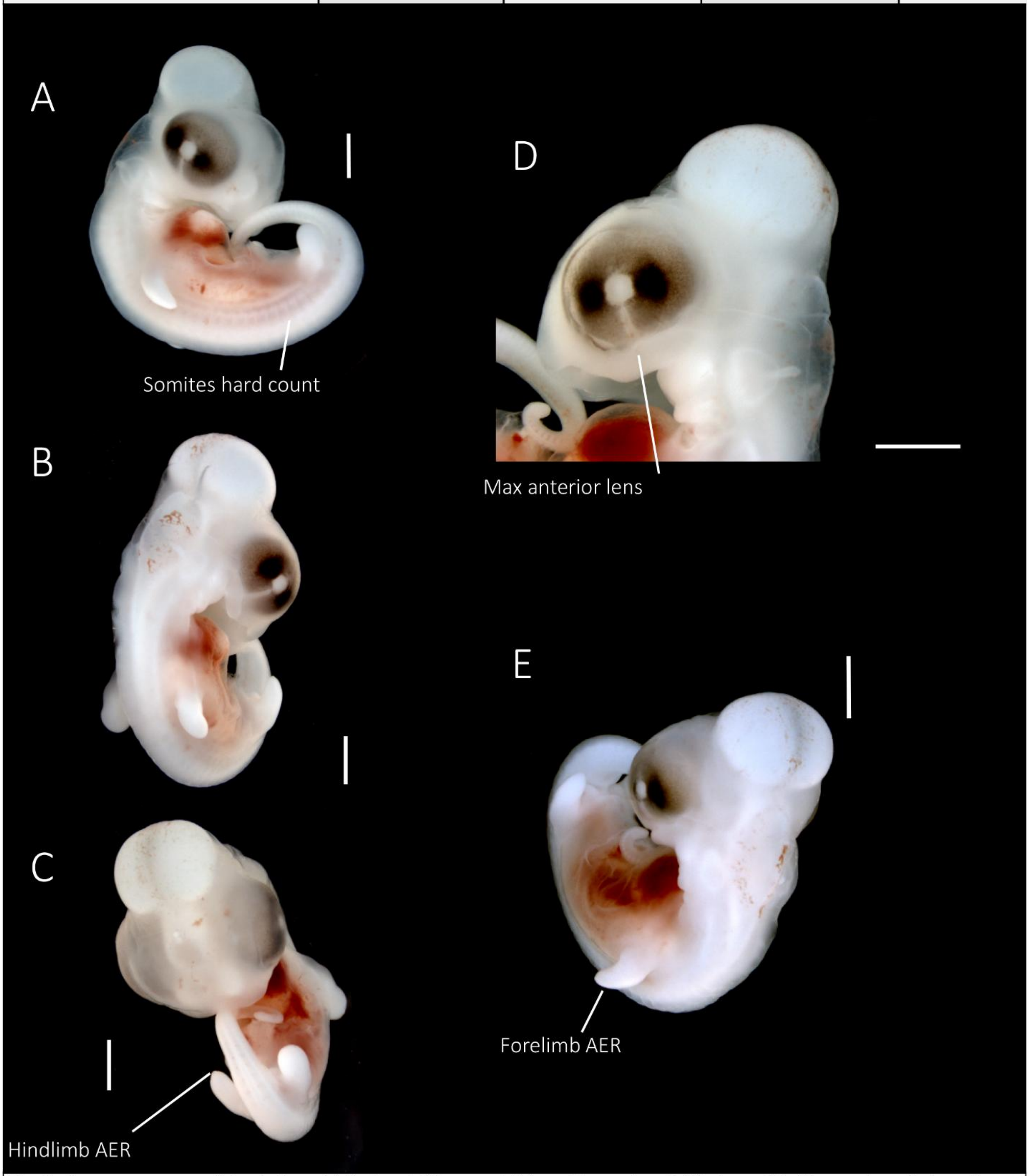

Fig. 3: *Pogona vitticeps* at 8 dpo in lateral (A), dorsal (B), ventral (C), and latero-dorsal (E) views. (D) Close-up lateral view of cranial and pharyngeal regions. Scale bars = 1 mm.

Standard Event System for Vertebrate Embryology

species (group)	stage/specimen	Specimen	specimen/stage No.	PV4
Pogona vitticeps	breeding temp.	29.5	collection No.	
	age (days)	12 dpo	sheet No.	5 / 11

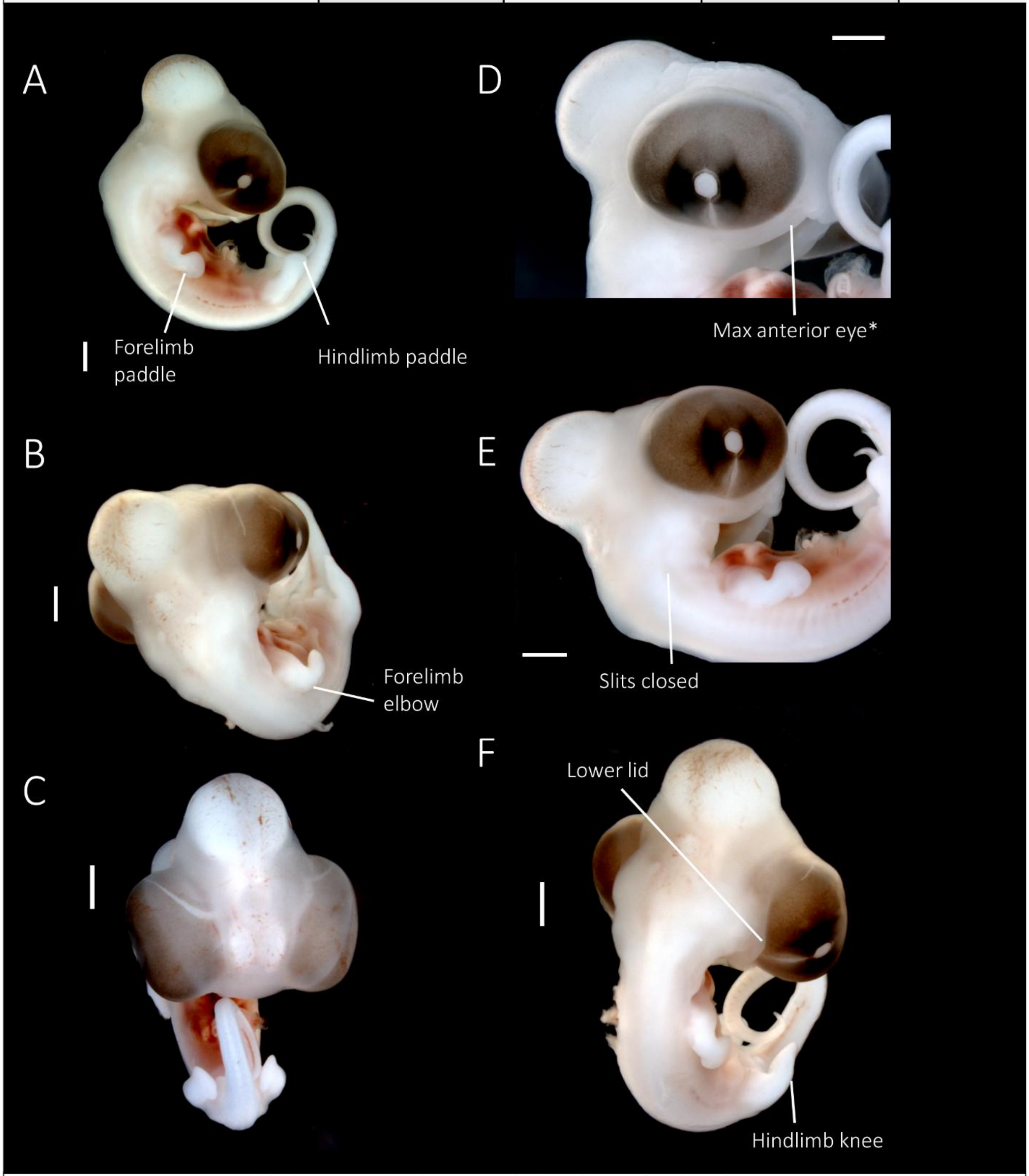

Fig. 4: *Pogona vitticeps* at 12 dpo in lateral (A), dorsal (B), ventral (C), and latero-dorsal (F) views. (D,E) Close-up lateral (D) and latero-dorsal (E) views of cranial region. Scale bars = 1 mm.

Standard Event System for Vertebrate Embryology

species (group)	stage/specimen	Specimen	specimen/stage No.	PV5
Pogona vitticeps	breeding temp.	29.5	collection No.	
	age (days)	15 dpo	sheet No.	6 / 11

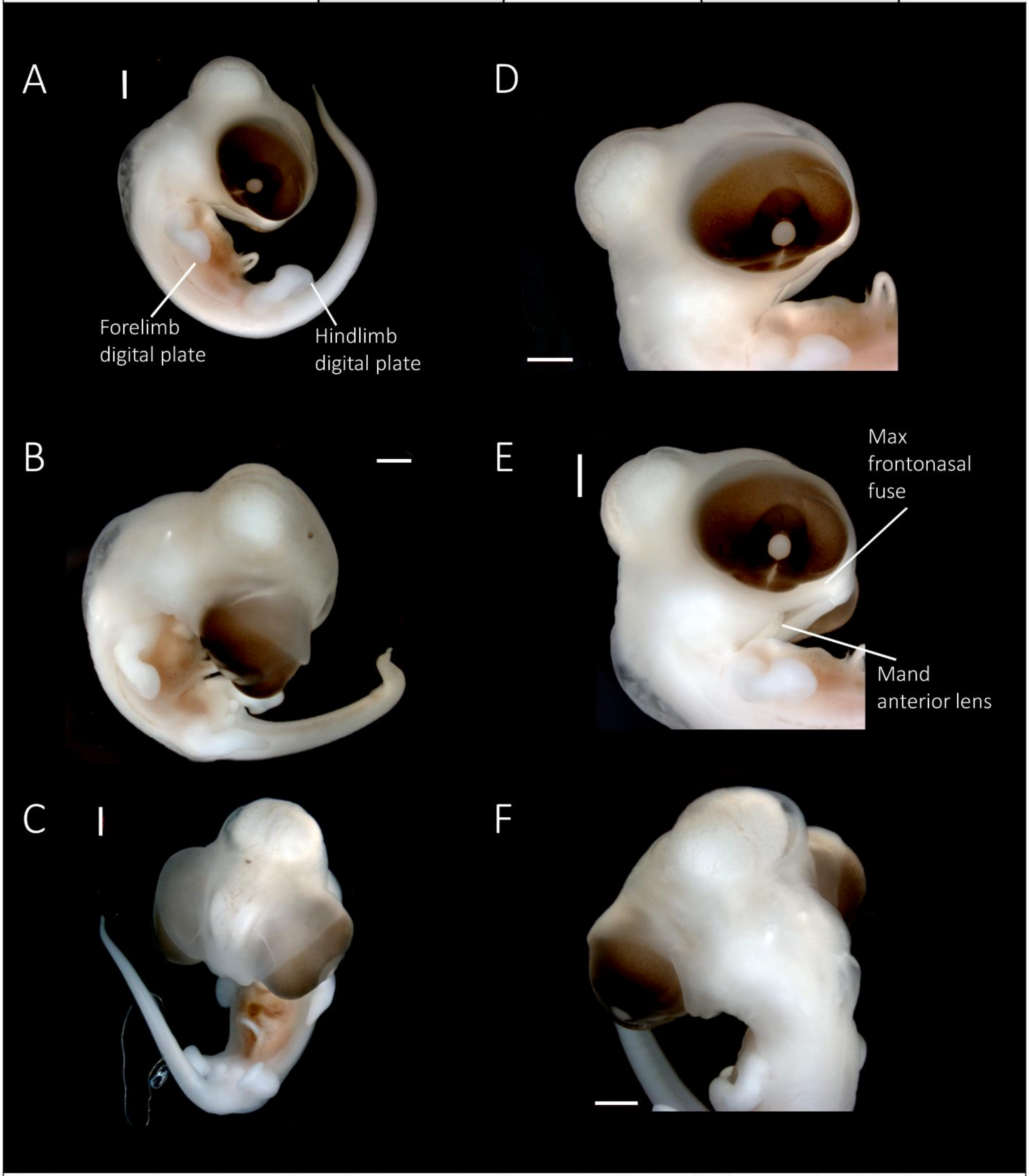

Fig. 5: *Pogona vitticeps* at 16 dpo in lateral (A), latero-ventral (B), and ventral (C) views. (D-F) Close-up lateral (D,E) and latero-dorsal (F) views of cranial region. Scale bars = 1 mm.

Standard Event System for Vertebrate Embryology

species (group)	stage/specimen	Specimen	specimen/stage No.	PV6
Pogona vitticeps	breeding temp.	29.5	collection No.	
	age (days)	18 dpo	sheet No.	7 / 11

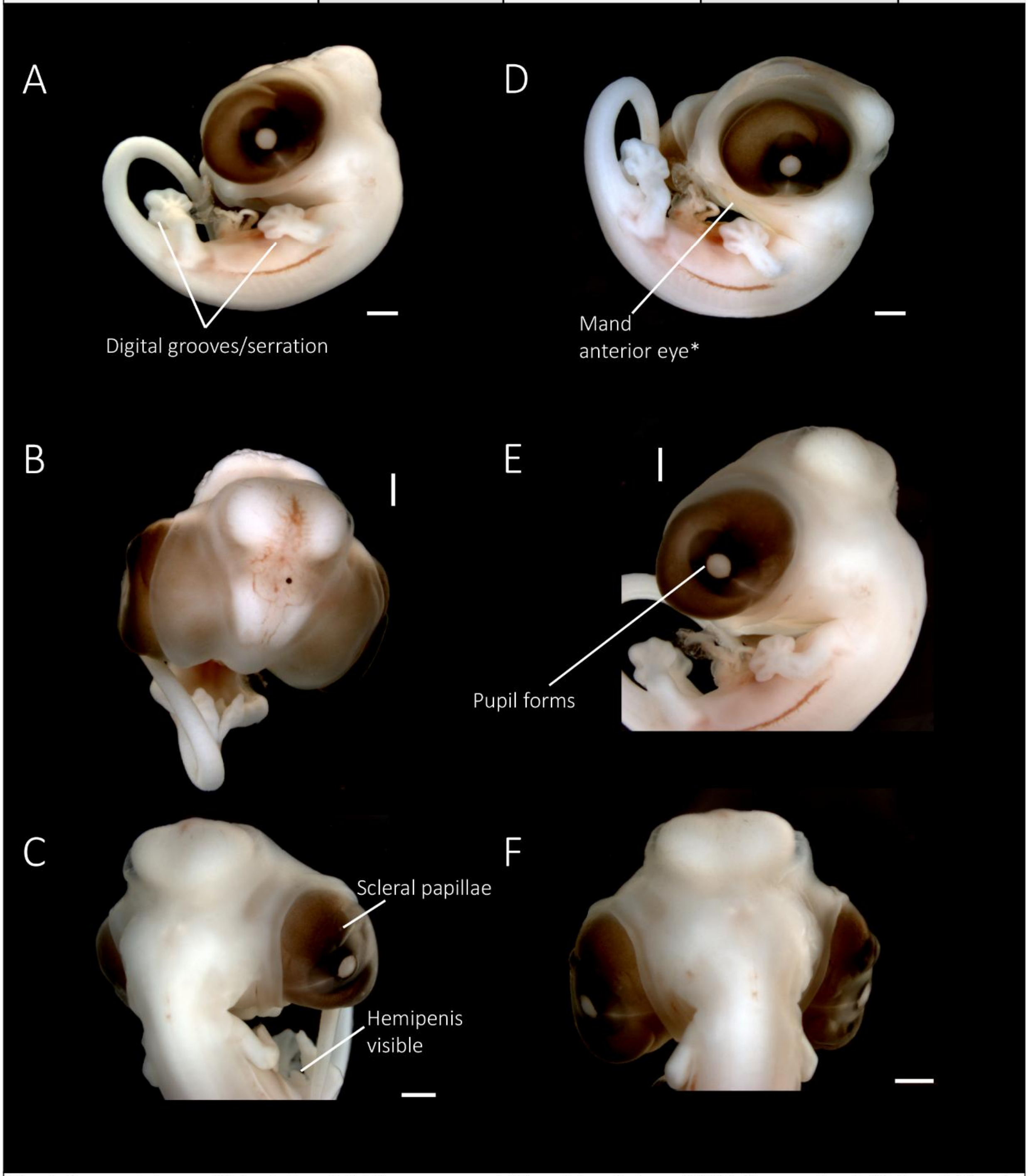

Fig. 6: *Pogona vitticeps* at 18 dpo in lateral (A,D), antero-ventral (B), and dorsal views. (C,E,F) Close-up dorsal (C), lateral (E) and antero-dorsal (F) views of cranial region. Scale bars = 1 mm.

Standard Event System for Vertebrate Embryology

species (group)	stage/specimen	Specimen	specimen/stage No.	PV7
Pogona vitticeps	breeding temp.	29.5	collection No.	
	age (days)	20 dpo	sheet No.	8 / 11

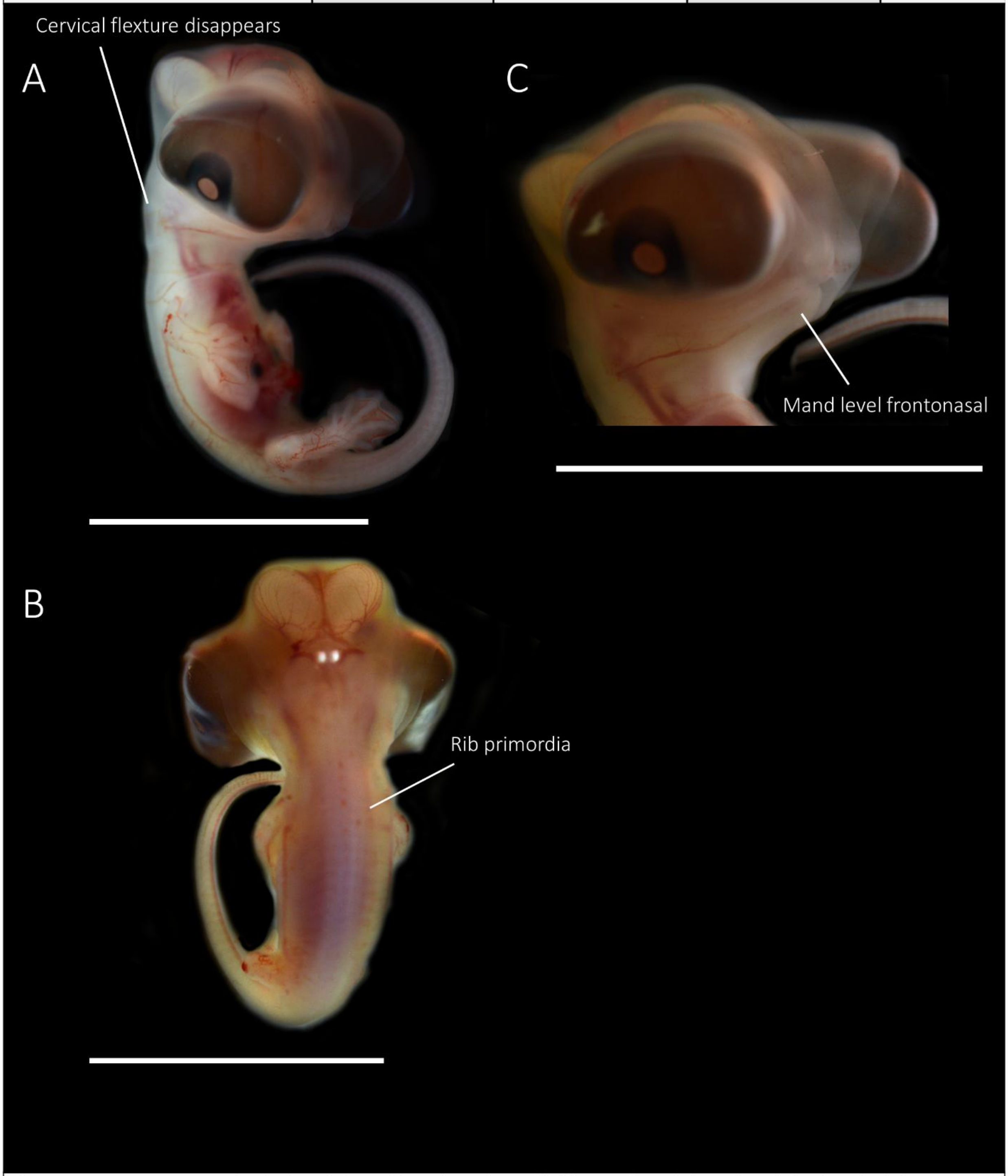

Fig. 7: *Pogona vitticeps* at 20 dpo in lateral (A) and dorsal (B) views. (C) Close-up lateral view of cranial region. Scale bars = 1 cm.

Standard Event System for Vertebrate Embryology

species (group)	stage/specimen	Specimen	specimen/stage No.	PV8-9
Pogona vitticeps	breeding temp.	29.5	collection No.	
	age (days)	24-28 dpo	sheet No.	9 / 11

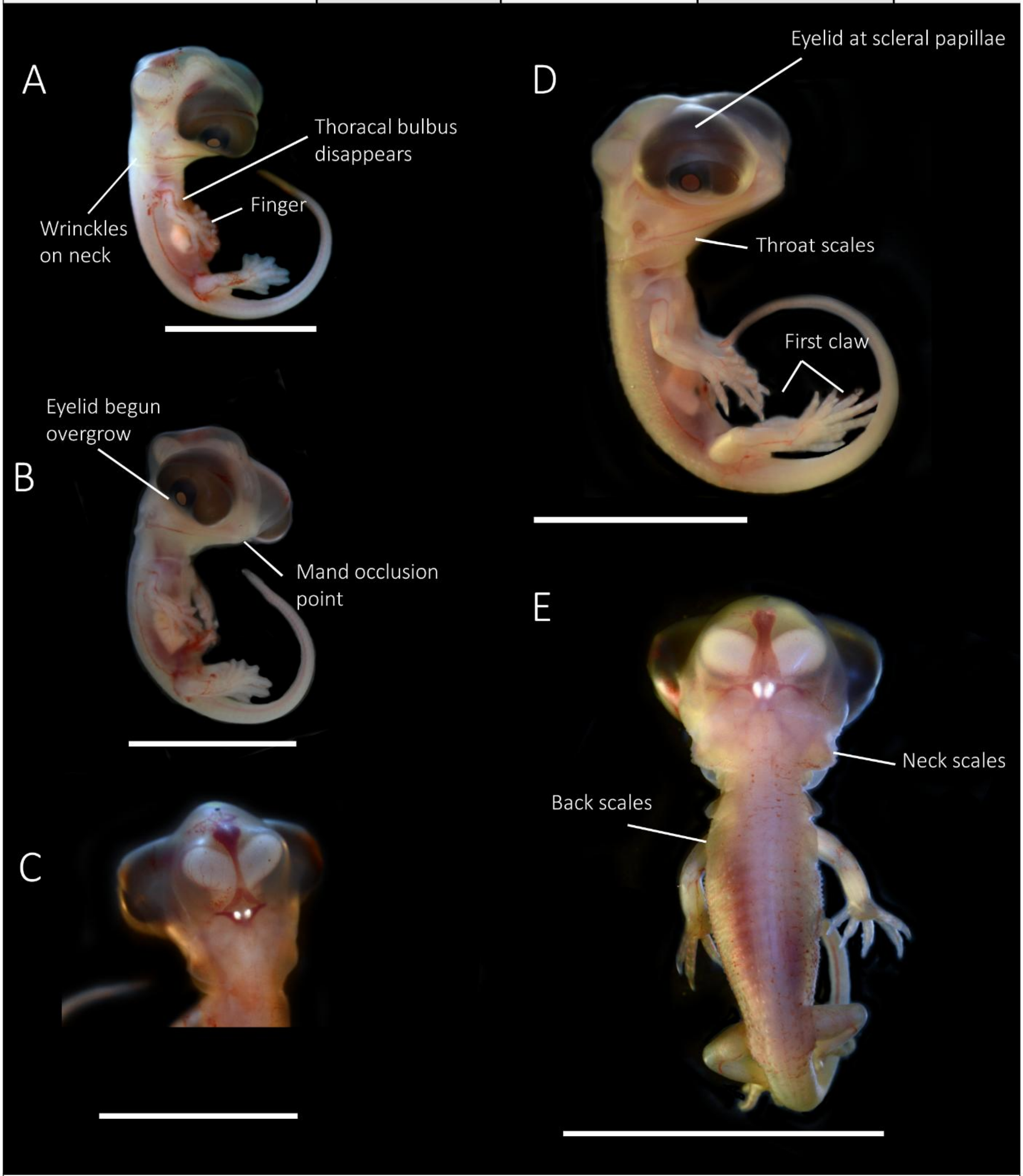

Fig. 8: (A-C) *Pogona vitticeps* at 24 dpo in lateral (A), ventro-lateral (B), and dorsal (C) views. (D,E) *Pogona vitticeps* at 28 dpo in lateral (D) and and dorsal (E) views. Scale bars = 1 cm.

Standard Event System for Vertebrate Embryology

species (group)	stage/specimen	Specimen	specimen/stage No.	PV10-11
Pogona vitticeps	breeding temp.	29.5	collection No.	
	age (days)	32-36 dpo	sheet No.	10 / 11

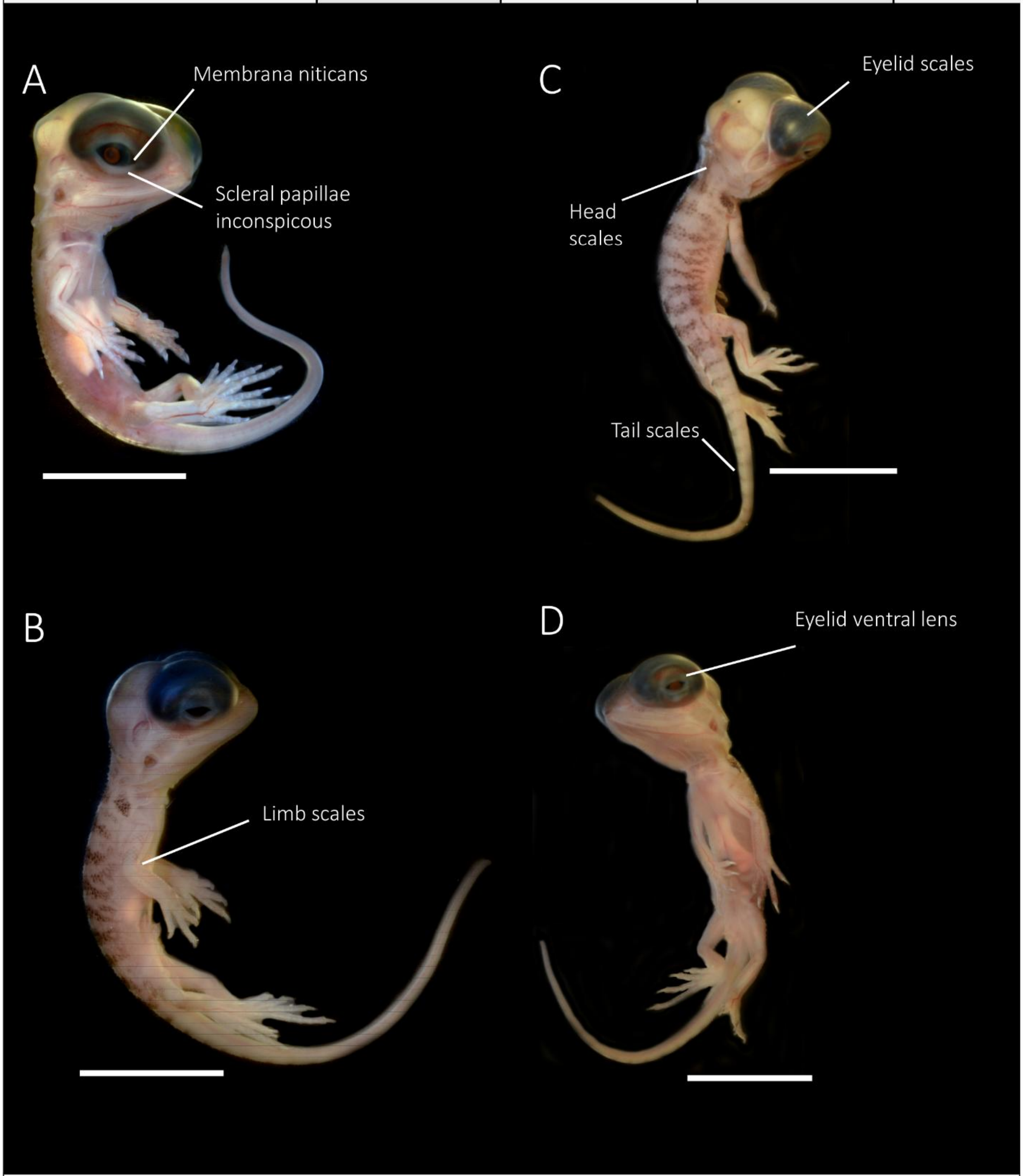

Fig. 9: (A) *Pogona vitticeps* at 32 dpo in lateral view. (B-D) *Pogona vitticeps* at 36 dpo in lateral (B), dorso-lateral (C), and ventral (D) views. Scale bars = 1 cm.

Standard Event System for Vertebrate Embryology

species (group)	stage/specimen	Specimen	specimen/stage No.	PV12-13
Pogona vitticeps	breeding temp.	29.5	collection No.	
	age (days)	40-60 dpo	sheet No.	11 / 11

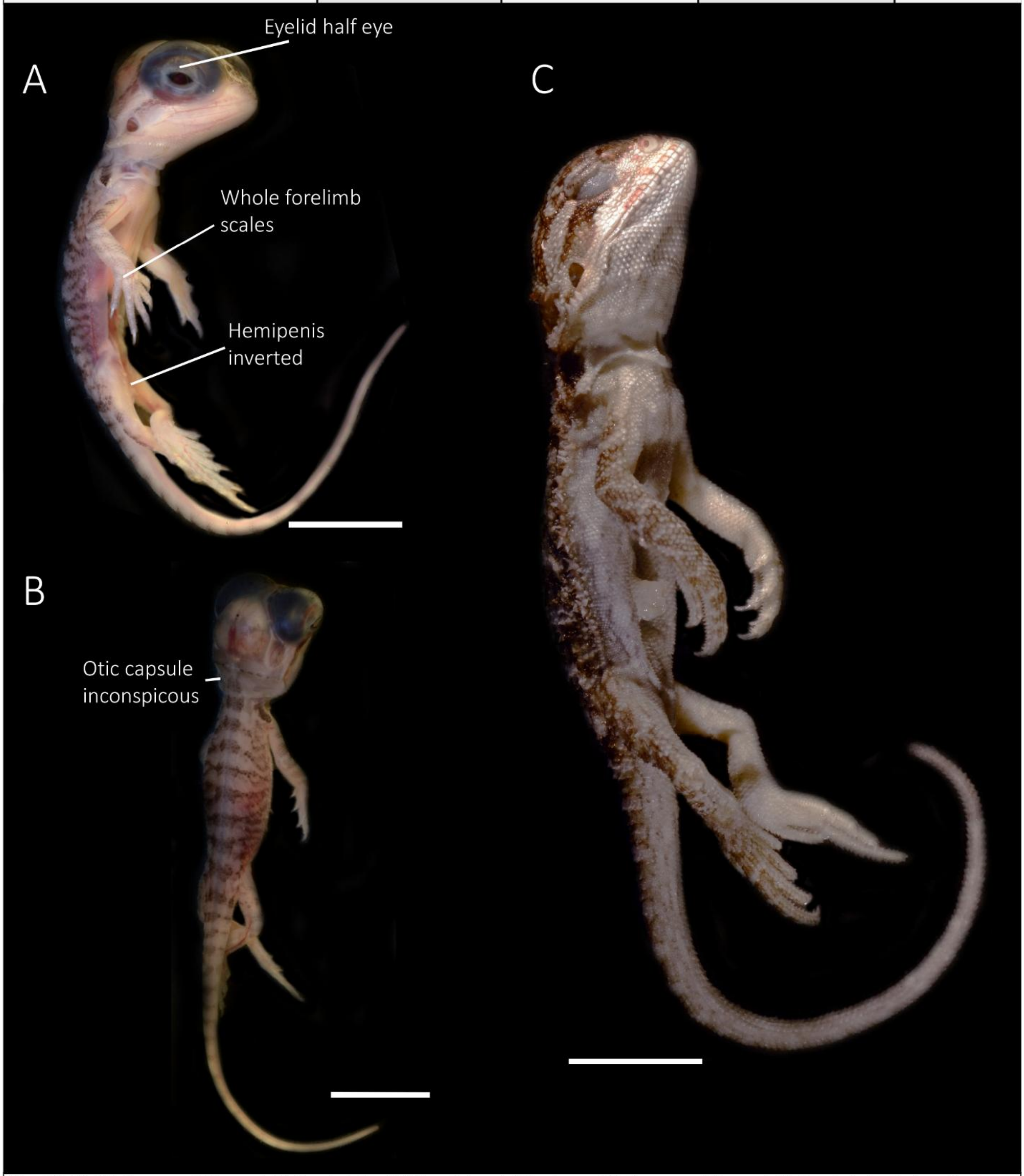

Fig. 10: (A,B) *Pogona vitticeps* at 40 dpo in lateral (A) and dorsal (B) views. (C) *Pogona vitticeps* at 60 dpo in lateral view. Scale bars = 1 cm.
