# Supplementary material for: Skull Development, Ossification Pattern, and Adult Shape in the Emerging Lizard Model Organism Pogona vitticeps: A Comparative Analysis With Other Squamates
Source: Front Physiol. 2018 Mar 28;9:278. doi: 10.3389/fphys.2018.00278 (PMC5882870; doi:10.3389/fphys.2018.00278)

Additional file 4. (A,B) 3D landmark points on the skull of the lizard *Pogona vitticeps* in lateral (A), ventral (B, top panel), and dorsal (B, bottom panel) views. (C) Skull of the lizard *Pogona vitticeps* in posterior views, showing the 3D landmark points on the back of the skull. See Additional file 6 for a complete list of landmarks.

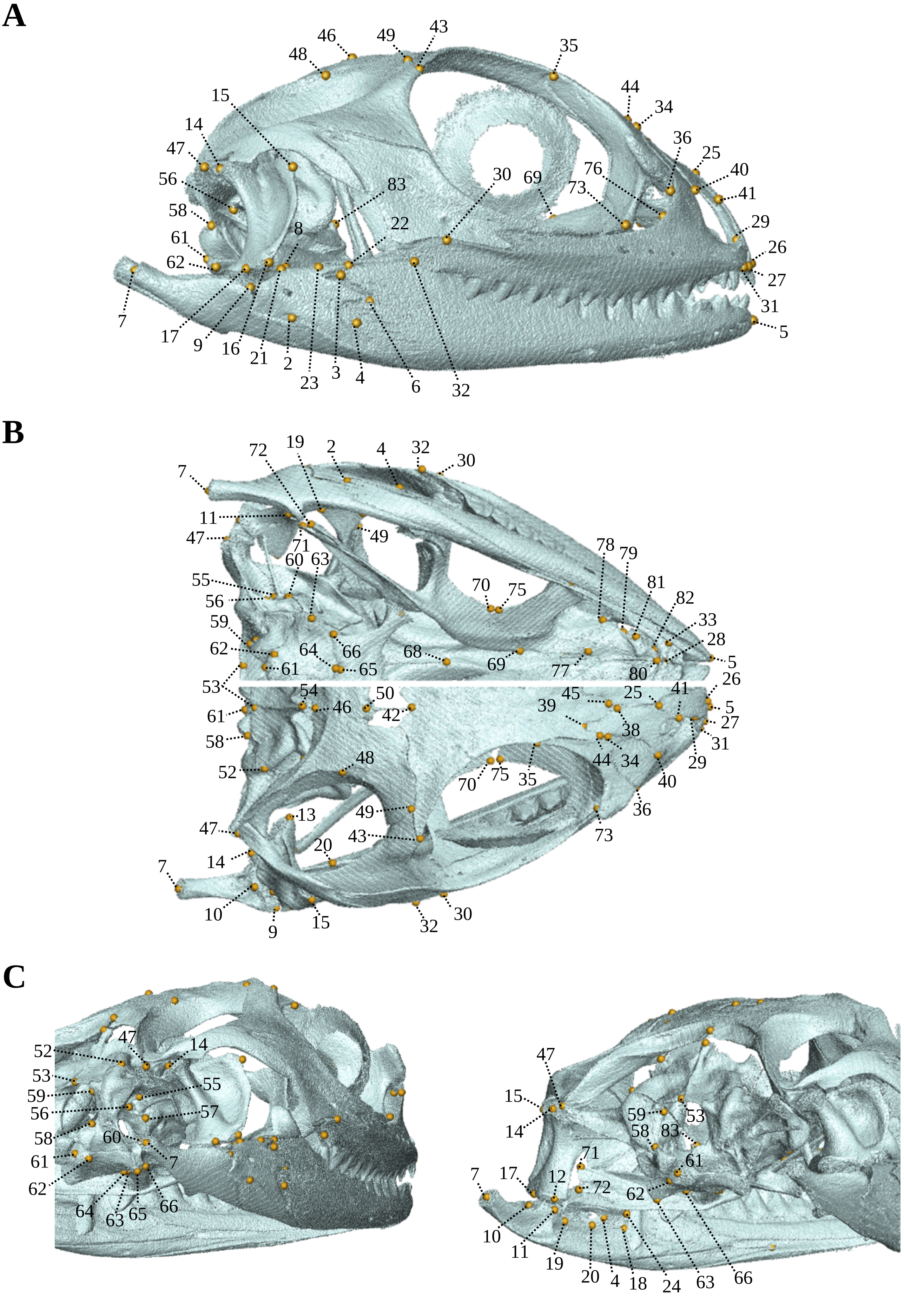

Supplement: Supplementary file 4 [file DataSheet4.pdf]
