# Supplementary material for: Skull Development, Ossification Pattern, and Adult Shape in the Emerging Lizard Model Organism Pogona vitticeps: A Comparative Analysis With Other Squamates
Source: Front Physiol. 2018 Mar 28;9:278. doi: 10.3389/fphys.2018.00278 (PMC5882870; doi:10.3389/fphys.2018.00278)
Supplement: Supplementary file 6 [file DataSheet6.PDF]

Additional file 6. Definition of 3D landmark points (see also Additional files 4 and 5).

Landmark number	Bone/region	Definition
L1	Angular/Splenial	Posterior tip of the angulo-splenial complex
L2	Splenial	Anterior tip of the angulo-splenial complex
L3	Dentary	Lateral tip of the dorsal process
L4	Dentary	Tip of the ventral process
L5	Dentary	Anterior tip of the dentary
L6	Dentary	Anterior inclination of the intramandibular hinge
L7	Articular/Compound	Posterior tip of the retroarticular process
L8	Articular/Compound	Anterior tip of the articular surface
L9	Articular/Compound	Lateral tip of the articular surface
L10	Articular/Compound	Posterior tip of the articular surface
L11	Articular/Compound	Medial tip of the articular surface
L12	Quadrate	Lateral tip of the ventral part of quadrate
L13	Quadrate	Anterior tip of the dorsal part of quadrate
L14	Quadrate	Medial tip of the dorsal part of quadrate
L15	Quadrate	Posterior tip of the dorsal part of quadrate
L16	Quadrate	Medial tip of the ventral part of quadrate
L17	Quadrate	Middle point of the ventral part of quadrate
L18	Articular/Compound	Anteriormost point of the prearticular process
L19	Articular/Compound	Posteriormost point of the preaticular process
L20	Articular/Compound	Dorsalmost tip of the prearticular process
L21	Surangular/Compound	Posteriormost point of the surangular process
L22	Surangular/Compound	Anteriormost visible point of the surangular process
L23	Surangular/Compound	Dorsalmost tip of the surangular process
L24	Surangular/Compound	Anteriormost border of fossa between surnagular and preaticular crests
L25	Premaxilla	Tip of the nasal process (see also L1 in Da Silva et al. 2018)
L26	Premaxilla	Rostral tip of the premaxilla (see also L2 in Da Silva et al. 2018)
L27	Premaxilla	Tip of the transverse process (see also L3 in Da Silva et al. 2018)
L28	Premaxilla	Medial point of the incisive process (lizards) or vomerine process (snakes) (see also L4 in Da Silva et al. 2018)
L29	Maxilla	Dorsal tip along the rostral margin of the premaxillary process (see also L6 in Da Silva et al. 2018)
L30	Maxilla	Dorsal tip along the posterior margin of the ectopterygoid process (see also L7 in Da Silva et al. 2018)
L31	Maxilla	Ventral tip along the rostral margin of the premaxillary process (see also L8 in Da Silva et al. 2018)
L32	Maxilla	Ventral tip along the posterior margin of the ectopterygoid process (see also L9 in Da Silva et al. 2018)
L33	Premaxilla	Tip of the transverse process (see also L10 in Da Silva et al. 2018)
L34	Prefrontal	Medial tip of the frontal process (see also L11 in Da Silva et al. 2018)
L35	Prefrontal	Lateral tip of the frontal process (see also L12 in Da Silva et al. 2018)
L36	Prefrontal	Tip of the prefrontal lateral foot process (see also L13 in Da Silva et al. 2018)
L37	Prefrontal	Rostral tip of the prefrontal outer wall (see also L14 in Da Silva et al. 2018)
L38	Nasal	Posterior tip of the medial margin of the nasal bone (see also L15 in Da Silva et al. 2018)
L39	Nasal	Posterior tip of the lateral margin of nasal bone (see also L16 in Da Silva et al. 2018)
L40	Nasal	Tip of the premaxillary process of the nasal bone on the lateral side (see also L17 in Da Silva et al. 2018)
L41	Nasal	Tip of the premaxillary process (see also L18 in Da Silva et al. 2018)

L42	Frontal	Medial point of intersection of the fronto-parietal suture (see also L20 in Da Silva et al. 2018)
L43	Frontal	Lateral tip of the fronto-parietal suture (see also L21 in Da Silva et al. 2018)
L44	Frontal	Anterior tip of the lateral margin of frontal bone (see also L22 in Da Silva et al. 2018)
L45	Frontal	Tip of the fronto-nasal suture (see also L23 in Da Silva et al. 2018)
L46	Parietal	Medial tip of the groove between the parietal bifid supraoccipital processes (see also L25 in Da Silva et al. 2018)
L47	Parietal	Tip of the parietal bifid supraoccipital process (see also L26 in Da Silva et al. 2018)
L48	Parietal	Tip of the postparietal process (see also L27 in Da Silva et al. 2018)
L49	Parietal	Lateral tip of the fronto-parietal suture (see also L28 in Da Silva et al. 2018)
L50	Parietal	Medial point of intersection of the fronto-parietal suture on the parietal bone (see also L29 in Da Silva et al. 2018)
L51	Supraoccipital	Antero-lateral tip of the supraoccipital-prootic suture (see also L32 in Da Silva et al. 2018)
L52	Supraoccipital	Postero-lateral tip of the supraoccipital-paroccipital suture (see also L33 in Da Silva et al. 2018)
L53	Supraoccipital	Postero-medial tip of the supraoccipital bone
L54	Supraoccipital	Base of the processus ascendens of supraoccipital (lizards) or antero-medial tip of supraoccipital (snakes) (see also L34 in Da Silva et al. 2018)
L55	Opisthotic	Ventral end of the opisthotic-prootic suture on the dorsal margin of the fenestra ovalis (see also L35 in Da Silva et al. 2018)
L56	Opisthotic	Inflection point on the posterior curvature of fenestra ovalis (see also L36 in Da Silva et al. 2018)
L57	Opisthotic	Dorsal end of the opisthotic-prootic suture on the ventral margin of the fenestra ovalis (see also L37 in Da Silva et al. 2018)
L58	Exoccipital	Most dorsal point of the occipital condyle L40
L59	Exoccipital	Lateral end of the exoccipital margin facing the foramen magnum (see also L41 in Da Silva et al. 2018)
L60	Exoccipital	Inflection point on the curvature of excoccipital bone along the border of the foramen magnum (see also L42 in Da Silva et al. 2018)
L61	Opisthotic	Most ventral point along the opisthotic-prootic margin at the intersection or near the pterygoid suture (see also L38 in Da Silva et al. 2018)
L62	Basioccipital	Most posterior medial point of the basioccipital condyle (see also L44 in Da Silva et al. 2018)
L63	Basioccipital	Most lateral point of the basioccipital condyle (see also L45 in Da Silva et al. 2018)
L64	Basioccipital	Tip of the spheno-occipital tubercle (see also L47 in Da Silva et al. 2018)
L65	Basioccipital	Antero-medial tip of the suture between basiooccipital and parabasisphenoid bones (see also L48 in Da Silva et al. 2018)
L66	Parabasisphenoid	Tip of the sagittal crest (see also L49 in Da Silva et al. 2018)
L67	Parabasisphenoid	Tip of the basipterygoid process (see also L50 in Da Silva et al. 2018)
L68	Parabasisphenoid	Tip of the cutriform process of basioccipital bone
L69	Pterygoid	Most dorsal point of the suture between the pterygoid and palatine bones (see also L57 in Da Silva et al. 2018)
L70	Pterygoid	Most ventral point of the suture between the pterygoid and palatine bones (see also L58 in Da Silva et al. 2018)
L71	Pterygoid	Most dorsal point on the posterior tip of the quadrate process (see also L59 in Da Silva et al. 2018)
L72	Prefrontal	Most ventral point on the posterior tip of the quadrate process (see also L60 in Da Silva et al. 2018)
L73	Prefrontal	Most posterior point of the prefrontal medial foot process (see also L61 in Da Silva et al. 2018)
L74	Palatine	Medial tip of the posterior edge of pterygoid process
L75	Palatine	Lateral tip of the posterior edge of pterygoid process
L76	Palatine	Posterior tip of the anterior (dentigerous) process in snakes or anteriormost tip of the maxillary process of palatine in lizards
L77	Vomer	Medial tip of the palatine process of palatine
L78	Vomer	Lateral tip of the palatine process of palatine

L79	Vomer	Anterior edge of the closure of Jacobson's organ in snakes or anterior tip of the lateral edge of the vomer in lizards before fenestra vomeronasalis
L80	Vomer	Anterior tip of the vomer
L81	Vomer	Posterior tip of the fenestra vomeronasalis
L82	Vomer	Anterior tip of the fenestra vomeronasalis
L83	Prootic	Anterior tip of the suture between prootic and basioccipital bones
