# Supplementary material for: Skull Development, Ossification Pattern, and Adult Shape in the Emerging Lizard Model Organism Pogona vitticeps: A Comparative Analysis With Other Squamates
Source: Front Physiol. 2018 Mar 28;9:278. doi: 10.3389/fphys.2018.00278 (PMC5882870; doi:10.3389/fphys.2018.00278)

Additional file 7. Plots of principal component (PC) scores showing the cranium **(A)** and mandible **(B)** shape distribution of lizards (grey shading) and snakes (red shading). For the complete list of species (with full names) see Additional file 2.

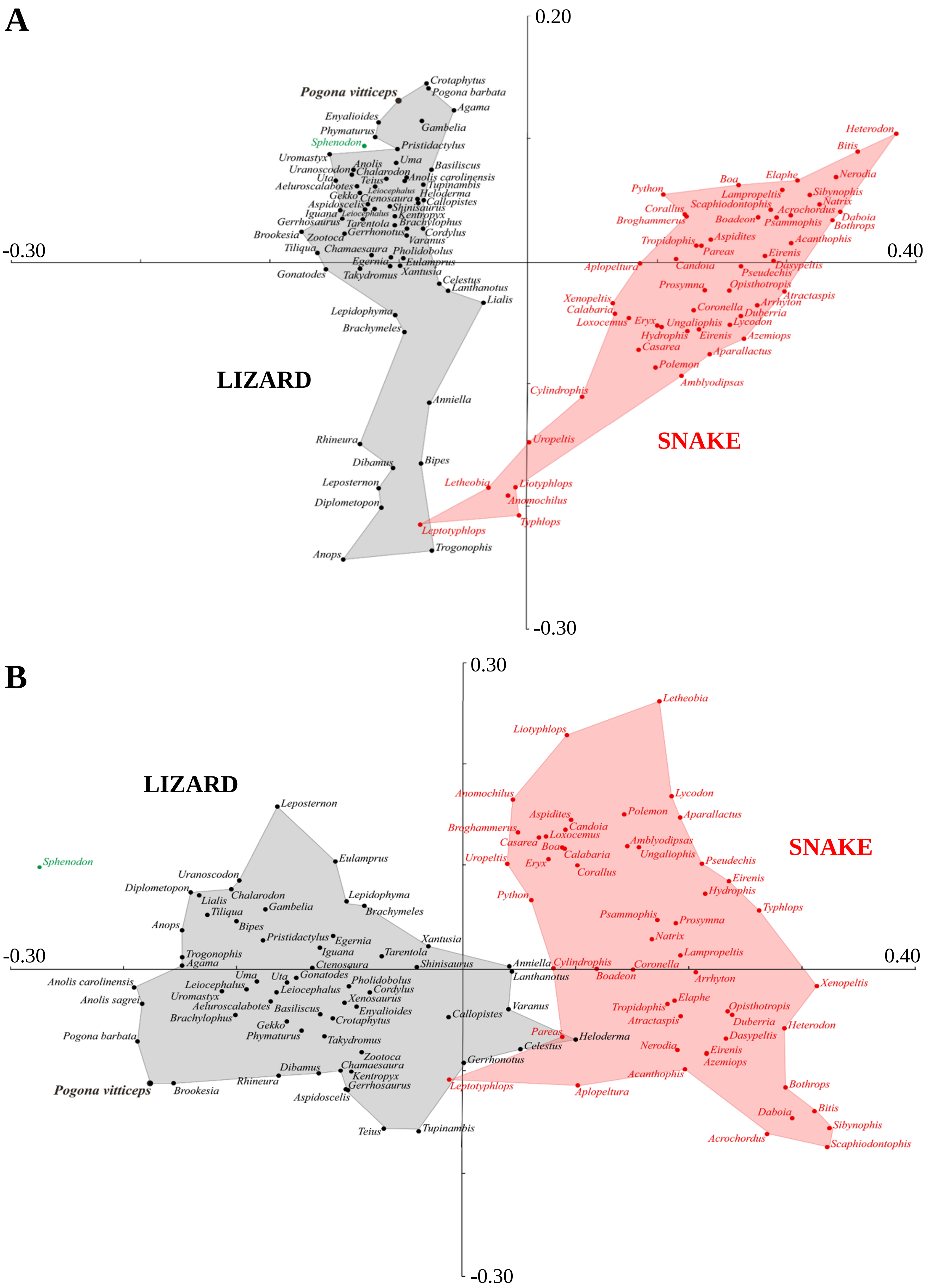

Supplement: Supplementary file 7 [file DataSheet7.pdf]
