# Supplementary material for: Skull Development, Ossification Pattern, and Adult Shape in the Emerging Lizard Model Organism Pogona vitticeps: A Comparative Analysis With Other Squamates
Source: Front Physiol. 2018 Mar 28;9:278. doi: 10.3389/fphys.2018.00278 (PMC5882870; doi:10.3389/fphys.2018.00278)
Supplement: Supplementary file 8 [file DataSheet8.pdf]

Additional file 8. Multivariate analysis of variance (MANOVA) with post hoc pairwise comparisons of cranium and mandible shape between major groups of squamates. *F*-values and *p*-values are shown in upper and lower off-diagonal cells, respectively. Significant values are highlighted in bold.

CRANIUM	Anguimorpha	Gekkota	Iguania	Lacertoidea	Scincoidea	Serpentes
Anguimorpha	-	2.71	3.5	1.41	**5.44**	**5.58**
Gekkota	0.1307	-	1.17	2.49	3.23	**11.95**
Iguania	0.0696	0.2882	-	**16.98**	**18.16**	**21.03**
Lacertoidea	0.2496	0.1344	**0.0002**	-	0.69	**5.25**
Scincoidea	**0.0351**	0.0996	**0.0002**	0.417	-	**7.07**
Serpentes	**0.0216**	**0.0011**	**<0.0001**	**0.0253**	**0.0101**	-

MANDIBLE	Anguimorpha	Gekkota	Iguania	Lacertoidea	Scincoidea	Serpentes
Anguimorpha	-	0.21	**16.25**	1.20	2.34	**7.70**
Gekkota	0.6533	-	**8.73**	1.01	1.78	**4.32**
Iguania	**0.0004**	**0.0069**	-	2.55	**6.65**	**7.14**
Lacertoidea	0.2868	0.3309	0.1197	-	0.12	**7.93**
Scincoidea	0.1486	0.2092	**0.0155**	0.7296	-	**5.33**
Serpentes	**0.0074**	**0.0425**	**0.0089**	**0.0065**	**0.0246**	-
