# Supplementary material for: Skull Development, Ossification Pattern, and Adult Shape in the Emerging Lizard Model Organism Pogona vitticeps: A Comparative Analysis With Other Squamates
Source: Front Physiol. 2018 Mar 28;9:278. doi: 10.3389/fphys.2018.00278 (PMC5882870; doi:10.3389/fphys.2018.00278)
Supplement: Supplementary file 9 [file DataSheet9.pdf]

Additional file 9. Character coding of the degree of development of 6 external facial traits (indicated by colored panels) in squamate embryos at the oviposition stage (0-1 dpo). Increasing score number (0-2) reflect more advanced developmental level. Arrows indicate the position of frontonasal prominence (fnp), maxillary prominence (mxp), oral commissure (oc), mandibular basal constriction (mac), mandibular prominence (map), and pharyngeal pouches (pp) on *Pogona vitticeps* embryo at 0 dpo.

I: Frontonasal prominence (fnp)

0 (not compressed)

1 (compressed, median and lateral prominences visible)

II: Maxillary prominence (mxp)

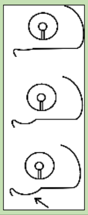

0 (not prominent or small)

1 (distinguishable, posterior to eye)

2 (large, anterior to eye)

III: Oral commissure (oc)

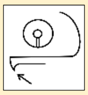

0 (ventral inflection)

1 (no)

IV: Mandibular basal constriction (mac)

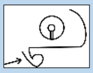

0 (presence)

1 (absence)

V: Mandibular prominence (map)

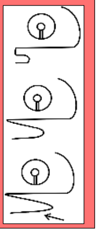

0 (posterior to eye)

1 (at the level of eye)

2 (anterior to eye)

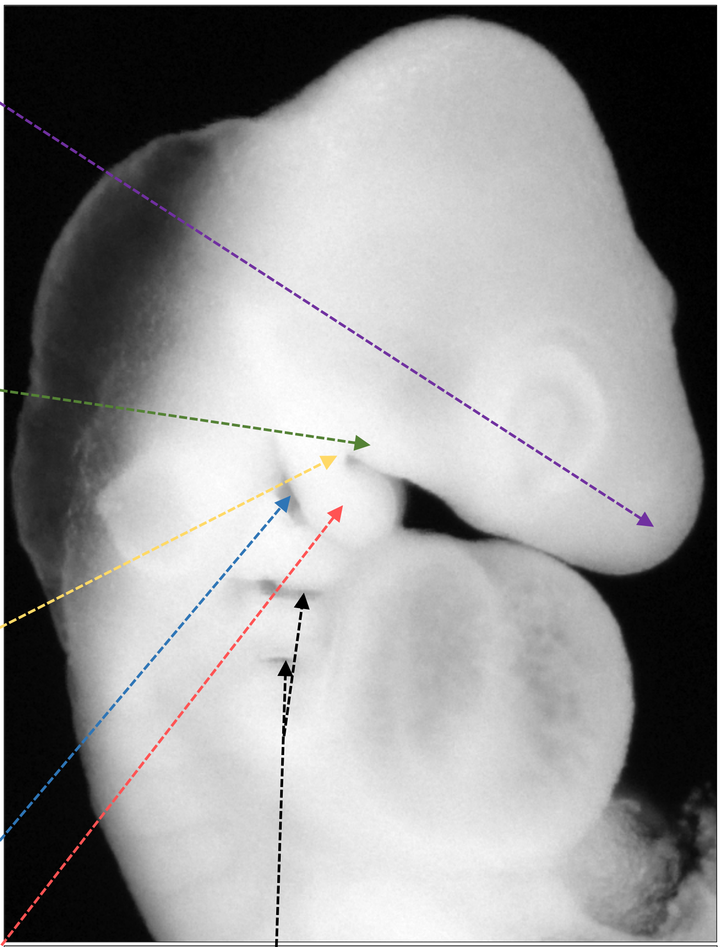

VI: Pharyngeal pouches (pp)

0 (not fused)

1 (partly fused)

2 (fused)
